# Gazing Into Language Development: Exploring Individual Variability in Early Word Recognition in Infancy Through Eye‐Tracking

**DOI:** 10.1111/infa.70028

**Published:** 2025-06-26

**Authors:** Anton Gerbrand, Johan Wengman, Linda Forssman

**Affiliations:** ^1^ Department of Psychology Uppsala University Uppsala Sweden

**Keywords:** CDI, early word recognition, eye tracking, infancy, language development

## Abstract

Previous research suggests that early word recognition is an important foundation for subsequent vocabulary development. However, the optimal method for assessing this ability in infancy remains unclear. To investigate this issue, we collected data from 70 participants (45.7% female) at 10, 11.5, 18 and 24 months of age using two eye‐tracking based tasks—the preferential looking‐ and mismatch paradigms—as well as parental reports on a short form of the Swedish Early Communicative Development Inventories (SE‐CDI). Both eye‐tracking‐based paradigms correlated with concurrent and later vocabulary scores. However, while the preferential looking paradigm showed stability across time, the mismatch paradigm did not demonstrate longitudinal stability and its associations with vocabulary were sometimes in unexpected directions. These findings suggest that the mismatch paradigm may reflect shifting cognitive or attentional processes during development, highlighting the need for further investigation. In contrast, the eye‐tracking based preferential looking paradigm, may offer an objective complement to parental reports for predicting subsequent vocabulary development in early childhood.

## 1 Introduction

Early word recognition is an important component of linguistic development (e.g., Bergelson 2020; Meylan and Bergelson 2022; Bornstein and Putnick 2012). Research indicates that children as young as 6–9 months can recognize familiar words (Bergelson and Swingley 2012; Parise and Csibra 2012; Tincoff and Jusczyk 1999), showcasing their ability to associate verbal labels with objects. This ability is believed to facilitate future word learning (Bergelson 2020; Bornstein and Putnick 2012; D'Souza et al. 2017) and the onset of word production at around 12 months (Fenson et al. 2000; Woodward et al. 1994).

Understanding language development during the first 2 years of life requires examining how early word recognition is associated with later vocabulary acquisition, including both word comprehension and production (Bornstein and Putnick 2012; Sander‐Montant et al. 2023). A key challenge involves finding methods that effectively capture individual differences in this early word recognition development.

Current approaches for assessing early word recognition and vocabulary, such as parental reports or gaze‐based observational methods, have limitations. Although vocabulary checklists like the MacArthur‐Bates Communicative Development Inventory (CDI), exhibit good test‐retest reliability and validity for children aged 8–30 months (Eriksson et al. 2002; Fenson et al. 2000; Jahn‐Samilo et al. 2001), parental reports may not adequately measure individual differences in pre‐verbal infants' word recognition. Parents may unintentionally overestimate or underestimate their child's abilities because these reports often involve interpreting subtle behavioral cues (Golinkoff et al. 2013).

By contrast, observational methods that record children's gaze allocation can provide more objective measures of early word recognition (e.g., Fernald et al. 2008; Swingley 2009). However, the reliability and validity of these paradigms as measures of individual differences in early language development remain to be fully established. One common observational method is the preferential looking paradigm (also known as the *looking‐while‐listening task;* Fernald et al. 2008). In this paradigm, children view two images (e.g., a banana and a pacifier) while hearing a prompt such as, “Where is the *pacifier*?” Word recognition is inferred when the child fixates longer on the target object.

A study using an eye‐tracking version of the preferential looking paradigm showed that infants as young as 6–9 months can recognize familiar words (Bergelson and Swingley 2012). Interestingly, this study found no concurrent correlation between infants' performance and parental CDI reports, with many parents reporting their children knew no words. This discrepancy suggests that parents may underestimate their child's early word recognition abilities (e.g., Bergelson and Swingley 2012). Another study with 12‐months‐olds (Lany et al. 2018) reported modest correlations between word recognition and concurrent CDI word comprehension percentile scores, as well as subsequent word production percentile scores around 15 months. These correlations, however, emerged only under certain conditions (i.e., “easy words” assessed within an early time window of each trial).

Studies with toddlers also show mixed results. While some studies find that preferential looking performance in the second year of life correlates with both concurrent and later vocabulary (Donnelly and Kidd 2020; Fernald and Marchman 2012; Marchman et al. 2016), others do not (Houston‐Price et al. 2007; Venker et al. 2016). These inconsistencies highlight the need for further research into how reliably early word recognition reflects individual differences in language development.

Another method, the mismatch paradigm (or word‐picture priming paradigm), is frequently used in EEG‐based research to study early word recognition (Junge et al. 2021). In this paradigm, participants hear a verbal label (e.g., “duck”) followed by a congruent or incongruent visual stimuli. Event‐related potentials (ERPs) measurements have shown that 9‐month‐olds can detect mismatches between verbal labels and visual stimuli, indicated by an increased N400 effect on incongruent trials (Parise and Csibra 2012). Notably, this effect emerged only when the infant's own mother provided the label, rather than the experimenter. Our study extends this approach by examining infants' pupil dilation during incongruent versus congruent trials, as changes in pupil size can signal mismatch detection (Kloosterman et al. 2015; Kuipers and Thierry 2011). The underlying assumption is that infants who have established word‐object associations will detect mismatches, reflected in increased pupil dilation during incongruent trials.

Although both the preferential looking and mismatch paradigms show that young infants recognize the meaning of familiar words, the link between early word recognition (as measured by these paradigms) and subsequent vocabulary, indexed by instruments like the CDI, remains largely unexplored. While it is reasonable to hypothesize that early task performance correlates with later vocabulary outcomes, there is currently insufficient evidence to confirm this link. It also remains unclear whether any observed associations represent stable, reliable individual differences in infancy, or if performance on these paradigms primarily reveal a group‐level phenomena, with meaningful individual differences developing only in toddlerhood. Should these paradigms capture reliable individual differences in infancy, they should correlate to later vocabulary, strengthening their utility as tools for assessing early language development, complementing parental reports.

Moreover, no research to date has directly compared performance in the preferential looking and mismatch paradigms, which appear to measure early word recognition through distinct mechanisms—gaze patterns and physiological responses, respectively. Investigating whether mismatch and preferential looking performance aligns and predicts later vocabulary outcomes is essential for validating their roles in measuring early language development.

### 1.1 Purpose of the Present Study

This study investigates the developmental stability of early word recognition using eye‐tracking assessments and its associations to concurrent and later vocabulary development, as measured by the Swedish Early CDI (SE‐CDI) short form. In a longitudinal design, we employed two eye‐tracking paradigms—the preferential looking and mismatch paradigms—alongside parental reports to examine children's word recognition and vocabulary development at 10, 11.5, 18 and 24 months of age. These time points were chosen to capture important developmental transitions: the word comprehension “boost” (Bergelson 2020) and start of word production around the end of the first year, and the rapid growth in both areas during the second year (Fenson et al. 2000; Woodward et al. 1994). Given the novelty of this research, we adopted a preregistered exploratory analysis approach to examine our hypotheses.

First, we addressed two primary research questions:

1. Is performance in the preferential looking and mismatch paradigms stable over time?
2. Is performance in the preferential looking paradigm related to performance in the mismatch paradigm?

We also explored two secondary research questions:

3 Do preferential looking and mismatch paradigm performance show concurrent and later association with vocabulary development at 10, 11.5, 18 and 24 m?
4 Are these associations influenced by socio‐demographic variables previously linked to vocabulary development?

Specifically, we will control for child gender (Hoff 2013), family socio‐economic status (SES; Piot et al. 2022), parental reading habits (Dong et al. 2020), monolingual status (De Houwer et al. 2006), family history of language problems (Bishop et al. 2012) and child cognitive functioning (Marchman and Fernald 2008; Peter et al. 2019).

## 2 Methods

### 2.1 Participants and Study Design

This study is part of the REaL cohort, a longitudinal project conducted in Uppsala, Sweden, following children from 10 to 24 months of age. The project consisted of two studies: (1) a longitudinal study (*N* = 70) that was started in September 2019 and ended in July 2021, and (2) a randomized controlled study with a longitudinal design (*N* = 115) registered as ISRCTN22319305 (Forssman and Gottwald 2022), which began in January 2020 and ended in October 2022.

Data collection in both two studies occurred at four time points, with the first three assessments involving lab visits. At all four time points, the participating parent completed an online questionnaire via a secure online platform (https://sv.surveymonkey.com). The current study is based on data collected in the longitudinal study at 10, 11.5, 18 and 24 months of age. The target sample size for this study was 70 children and their families. The sample size was determined prior to enrollment, based on practical considerations such as the availability of participants within the target age range, the study timeline, and the resources required for labor‐intensive laboratory assessments. Table 1 presents a socio‐demographic description of the sample.

**TABLE 1:** Socio‐demographic description of sample (*N* = 70).

	% (*n*)	*M* (SD)	Min–max
Child age in days			
Time 1, 10 months		309.41 (17.28)	273–339
Time 2, 11.5 months		356.91 (19.46)	316–395
Time 3, 18 months		545.42 (17.91)	519–584
Time 4, 24 months		737.48 (12.21)	715–789
Child female gender^a^	45.71 (32)		
Participating parent female gender (mother)^a^	74.29 (52)		
Parental education^b^			
Participating parent			
No university education	12.9 (9)		
University education (≥ 3 years)	84.3 (59)		
Other parent			
No university education	24 (17)		
University education (≥ 3 years)	70 (49)		
Swedish language spoken at home^a^			
< 100% of the time	24.28 (17)		
100% of the time	75.71 (53)		
Reading habits at home^b^ ^,^ ^c^		0.00 (0.89)	−2.03 to 1.68
Family history of language delay or language disorder^b^			
Yes	15.71 (11)		
No	81.43 (57)		

^a^ Assesed during the screening process.

^b^ These questions were asked in relation to the first lab visit when the child was 10 months old (response rate = 69/70).

^c^ Reading habits at home represent a standardized composite score of reading frequency and length of reading.

Children and their families were recruited using several methods: (i) by contacting volunteer families who had indicated a general interest in participating in studies at the Uppsala Child and Baby Lab; (ii) by inviting participants from a population‐based study in Uppsala, Sweden, investigating perinatal mental health (Axfors et al. 2019); (iii) by posting flyers about the study at family centers and open‐preschools in Uppsala; and (iv) by using social media (e.g., Twitter and Facebook).

**FIGURE 1:**
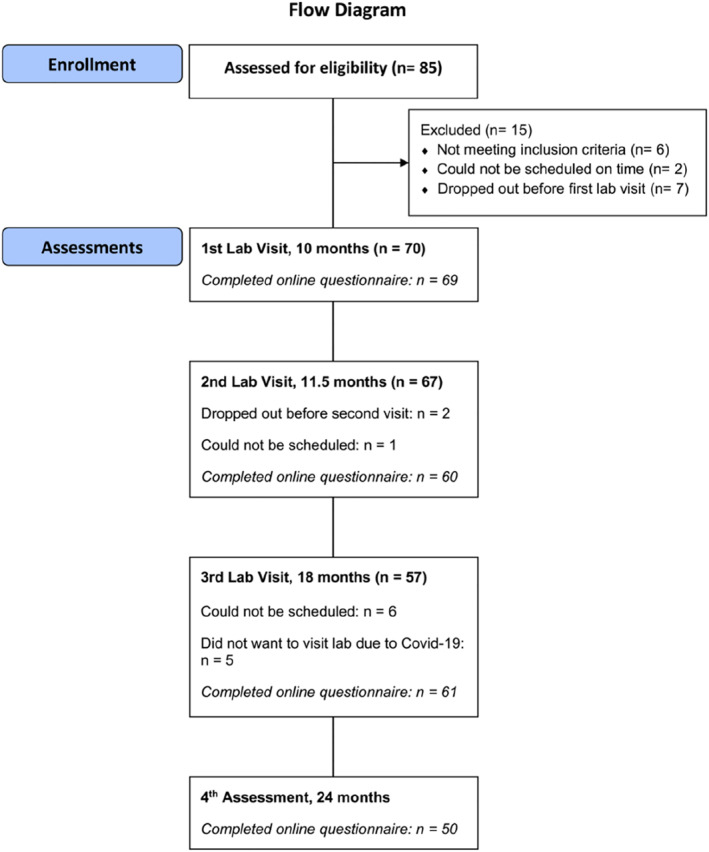
Diagram showing flow of participants through the study. This diagram shows flow of participants from screening assessment of eligibility to participate in the study to the fourth assessment at 24 months of age.

Prior to enrollment, parents (i.e., the child's legal guardians) who had received information about the study and indicated an interest in participating were screened for inclusion and exclusion criteria. Families were eligible to participate if they met all of the following inclusion criteria: (i) the child's parent(s) gave consent to study participation; (ii) the participating child was 10‐months‐old (± 4 weeks) at the time of the first assessment; (iii) the family spoke Swedish (at least some) at home; (iv) the same participating parent (mother or father) was able to attended the first two lab visits with their participating child (this was due to the inclusion of parent‐child interaction observations at these two assessment points). Families were not eligible to participate if they met any of the following exclusion criteria: (i) the child was born prematurely (less than 37 weeks of gestations) and/or (ii) the child had an illness or disability that could prevent them from fully participating in the study. Figure 1 provides a flow a diagram of participants throughout the study.

The research was approved by the Swedish Ethical Review Authority (Etikprövningsmyndigheten; reference number 2019‐03140) and conducted in full compliance with the Helsinki Declaration. Parents provided written consent prior to the start of the study and again at each subsequent visit. Participating families received a gift voucher (≈10 €) at each lab visit as form of travel reimbursement.

### 2.2 Measures and Procedures

The first two lab visits ranged from approximately 75–90 min (including breaks) and the third lab visit took approximately 60 min (including breaks). An online administrated questionnaire, sent approximately 1 week before each lab visit, took about 60 min to complete. The lab visits included direct child assessments with eye tracking (Tobii Pro TX300/Tobii X3‐120, Tobii technology, Stockholm), Electroencephalography system (EEG; 128 channel net, Electrical Geodesics Inc., EGI), video‐recorded observations, and a tablet test, as well as video‐recorded structured observations of parent‐child interaction. A full list of tasks used in the REaL project can be found at Databrary (Forssman 2022; https://nyu.databrary.org/volume/1506).

For this study, the primary outcome measures were assessments of children's word recognition, comprehension, and production, based on eye tracking tasks and parental reports. The study's outcome measures are presented in Table 2. Note that the mismatch paradigm was not available at 18 months due to earlier design considerations for the longitudinal project from which these data are drawn.

**TABLE 2:** Study outcomes and measures at 10‐month (T1), 11.5 months (T2), 18‐month (T3) and 24‐month (T4).

Outcomes	Measures	T1	T2	T3	T4
Word production	Parental report: SE‐CDI	x	x	x	x
Word comprehension	Parental report: SE‐CDI	x	x	x	x
Word recognition	Eye tracking: Preferential looking paradigm	x	x	x	
Word recognition	Eye tracking: Mismatch paradigm	x	x		
Covariates					
Cognition	Bayley cognitive scale	x			
Child gender	Parental report	x			
Parental education	Parental report	x			
Language spoken at home	Parental report	x			
Reading habits at home	Parental report	x			
Family history of language difficulties	Parental report	x			

Abbreviation: SE‐CDI = The Swedish Early Communication Development Inventory.

#### 2.2.1 Child Characteristics and Socio‐Demographic Data

Child characteristics and socio‐demographic data were obtained through the screening process and an online parental questionnaire. The following variables were included: child gender (boy/girl), parental education (parent has a university education: yes/no), language spoken in home (100% Swedish: yes/no), familial history of language difficulties (yes/no), and reading habits. The outcome measure for reading habits consisted of a composite z‐score of reading frequency and length of reading, where higher values indicate more reading activities. Parents reported the frequency with which they read to their child (never, 1–2 times per month, 1–2 time per week, or everyday/almost every day) and the amount of time they spent reading to their child on the previous day (0 min, 1–10 min, 11–20 min, more than 20 min).

#### 2.2.2 Child General Cognitive Functioning

General cognitive functioning was assessed at the first lab visit with the Bayley‐III Cognitive Scale (Bayley 2009). This measure estimates nonverbal problem‐solving, object manipulation, and memory. Children receives points for each completed task (e.g., manages to lift and move a small cube). The total score was converted to a standardized scaled score (Bayley 2009).

#### 2.2.3 Parental Reports on Child Vocabulary Development

At all four assessment points, parents completed the *Swedish Early Communication Development Inventory* (SE‐CDI; Eriksson et al. 2002) measuring *word comprehension* (number of words the child understands) and *word production* (number of words the child understands and say). This 90‐item checklist yields two aggregated scores (min–max = 0–90) for word comprehension and word production. CDI data were included only if parents had answered ≥ 90% of the items. Supporting Information S1: Table S1 provides details on missing data at each assessment point.

### 2.3 Eye Tracking Assessments of Word Recognition

Two eye tracking tasks were used to measure children's word recognition: *the preferential looking paradigm* and *the mismatch paradigm*. During these tasks, the child sat on a parent's lap ∼60 cm from 22″ screen. Gaze were collected at 120 Hz using a Tobii Pro TX300 during the first 73 lab visits and a Tobii X3‐120 thereafter (Tobii Technology AB, Stockholm, Sweden). A five‐point calibration was conducted prior to the start of each task. Trials continued until the end of each as long as the child attended to the screen; the session ended if the child became inattentive or fussy.

In both tasks, pre‐recorded sentences were presented. These were recorded by the same female speaker, a native Swedish speaker with a local accent. She used a child‐directed speech during recording, and the audio files were normalized for pitch and volume.

#### 2.3.1 Preferential Looking Paradigm

This paradigm (modeld on Bergelson and Swingley 2012) comprised 4 warm‐up trials followed by 32 test trials. Each trial began with a central fixation stimulus (a red cross) accompanied by a recorded sentence directing the child to look at a picture (e.g., “Look at the foot!”). The sentence length ranged from 550 to 1250 ms (mean: 1058 ms), always ending 250 ms before the presentation of the pictures.

**FIGURE 2:**
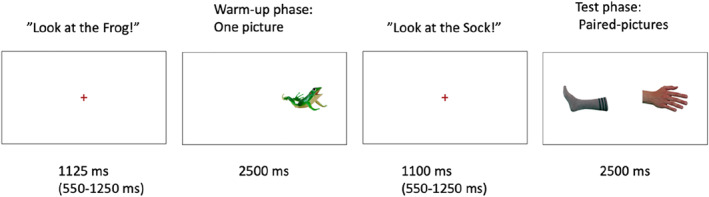
Experimental timeline for preferential looking paradigm. On each trial the child was presented with a fixation cross and a prerecorded sentence over speakers (e.g., “Look at the Frog!”). The sentences always ended 250 ms prior to the end of the presentation of the fixation cross. During the warm‐up phase (4 trials) the child was presented with a single picture on the screen (counterbalanced for left and right side). During the test phase the child was presented with pairs of pictures for 2500 ms. The side of the targeted picture was counterbalanced between left and right.

On warm‐up trials, a single picture (a sheep, a pig, a monkey, or a frog) appeared for 2500 ms to the left or the right of the center (alternating L, R, L, R). On test trials, pairs of pictures (target + distractor) appeared for 2500 ms on the left and right sides of the screen. Each pair (e.g., hand‐sock) was presented four times, twice with hand as the labeled target, twice with sock as the labeled target. Presentation order was pseudorandomized, such that the target picture could not be presented on the same side for more than one trial and could not be presented in two consecutive trials. See Supporting Information S1: Table S2 for all included words and objects. Child‐friendly video clips (8 s long) were presented after every 8th trial to maintain attention. The total administration time was ∼5 min. Parents were instructed not to assist their child (e.g., pointing to the screen). Figure 2 illustrates stimuli and trial sequences.

Looking time to the target and the distractor pictures was calculated within an analysis window from 367 to 2500 ms after the onset of the presentation of the pictures. This starting point accounts for the time children require to initiate an eye movement in response to the auditory signal (Bergelson and Swingley 2012). A trial was considered valid if the child provided at least 30% (∼640 ms) gaze data during the analysis window. Children provided, on average, 22.05 (SD = 9.11), 25.08 (SD = 7.57) and 29.96 (SD = 4.02) valid trials (out of 32 trials) during the first, second and third lab visit, respectively. A proportion‐of‐looking‐time score for the target was calculated by subtracting looking time at the same picture when it functioned as a distractor. Thus, a positive score indicates word recognition (Bergelson and Swingley 2012). This approach controlled for any inherent bias toward one picture over another and produced a single score per item pair. The mean score across valid trials served as the outcome measure. Children had to provide valid data for at least four out the eight word‐pairs to be included in the analysis. On average, children provided 6.07 (SD = 2.43), 6.73 (SD = 2.08) and 7.92 (SD = 0.27) valid word‐pairs out of 8 at the first, second and third lab visit, respectively (see Supporting Information S1: Table S1 for information on percentage of missing data). Technical issues with the eye‐tracker at 10 and 11.5 months resulted in data loss for 7 participants.

#### 2.3.2 Mismatch Paradigm

**FIGURE 3:**
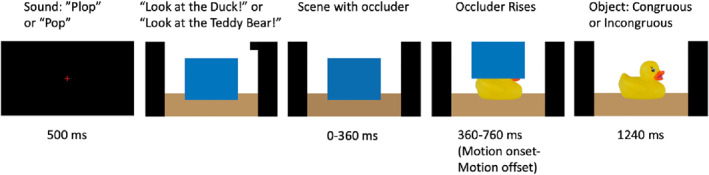
Experimental timeline for the mismatch paradigm. On each trial the child and the parent were presented with a fixation cross, and the parent heard a brief sound (“pop” or “plop”—alternating every second trial) over the headphones. Next, a scene with a blue occluder was presented on the screen while the parent heard a sentence over the headphones. As soon as the parent had repeated the sentence, the experiment leader pressed a button (the spacebar) on the experimenter computer. Following the button press, 360 ms later the occluder started to rise and revealed the object. By 760 ms the object was presented by itself on the screen and remained there for 1240 ms.

This paradigm (modeled on Parise and Csibra 2012) comprised 64 trials and included 16 words, each presented four times ‐ twice followed by a congruous picture and twice by an incongruous picture (see Supporting Information S1: Table S2 for a list of all words and objects used). To maintain the child's attention to the task, child‐friendly pictures and video clips were presented after every 4th trial. The task took ∼7 min to administrate. Figure 3 depicts the stimuli and trial sequences.

During this assessment, the child's parent wore headphones and heard a series of pre‐recorded (e.g., “Look at X!”), which they repeated verbatim to their child. Parents repeated the sentences naturally, as they would in everyday life, and could also use gestures, such as pointing, to direct the child's attention to the screen. Each trial began with a central 500 ms fixation stimulus (a red cross) at the center of the screen. During this phase the parent heard a short sound (a “pop” or a “plop”) signaling that a sentence would play next. Next, a scene with floor and a blue occluder was displayed. A sentence prompt (“Look at X!”) was presented over headphones to the parent, who repeated it to the child. Then the experimenter pushed a button, which initiated that the occluder moved upwards (motion onset = 360 ms, motion offset = 760 ms). A picture of an object was revealed for 1240 ms that was either congruent (50%) or incongruent (50%) with the parent's labeled word. Trials were pseudorandomized so that there could be no more than two consecutive congruent or incongruent trials, and the same word could not be presented more than twice in a row.

The outcome measure was a pupil size difference score derived by subtracting mean pupil size on incongruent trials from congruent trials Thus, a positive difference score reflects larger pupil size on incongruent trials. The score was baseline corrected (see e.g., Hepach and Westermann 2013) using a 360 ms interval prior to occluder movement. The 1200 ms time window that followed the occluder's disappearance served as the pupil dilation analysis window (Kuipers and Thierry 2011). Any individual trial missing more than 50% of data across the baseline and analysis window was excluded. To be included in the analyses, each child needed at least 5 valid trials in each condition (congruent and incongruent). On average, children who met these criteria provided 32.2 (SD = 15.1) trials and 36.7 (SD = 14.7) at the first and second lab visit, respectively (see Supporting Information S1: Table S1 for missing data). Technical issues with the eye‐tracker at 10‐ and 11.5‐month visits resulted in data loss for 12 participants.

### 2.4 Pre‐Processing of Eye Tracking Data

The raw gaze data files were exported from Tobii Studio and processed in TimeStudio (version: 3.23, http://timestudioproject.com; Nyström et al. 2016), operating within the MATLAB environment (version: R2022b; The Mathworks, Natick, MA). For the preferential looking paradigm, missing data segments shorter than 14 samples (117 ms) were linearly interpolated (Leppänen et al. 2015). Two areas of interest (AOIs) were defined, covering the target and distractor pictures (subtending 11° visual angel horizontally and 13.6° visual angle vertically off‐center; see Supporting Information S1: Figure S1). For the mismatch paradigm, pupil data were analyzed from the participant's left eye. Samples outside the 1.5–9 mm pupil size range were discarded, and a 14‐sample moving median average filter was applied. Short data gaps (≤ 14 samples) were linearly interpolated.

### 2.5 Statistical Analysis

All statistical analysis were performed in SPSS (version 28). The variables, outcome measures and main analyses were pre‐registered before any data analyses (see pre‐registration https://osf.io/4qekb/?view_only=7e303836d2d4480eab2f42fdea8e57f6) and both the data and analyses are available at https://osf.io/sbzrt/?view_only=b6d2cd11ade848ef80a39f438738795c.

Data was examined for non‐normality using Q‐Q plots, histograms, and skewness/kurtosis values. As the SE‐CDI and mismatch paradigm measures were not normally distributed and exhibited outliers with a large impact on the data, Spearman's rho was used for correlation matrixes. This deviated from the preregistration, which proposed transforming non‐normally distributed data; however, no standard transformation successfully normalized the SE‐CDI measures.

Missing data in the primary outcome measurements were handled by using the expectation‐maximization (EM) algorithm to facilitate statistical analysis of the full sample (*N* = 70). This procedure uses all relevant parameters in the data matrix, imputing missing values based on relationships among remaining variables. The EM algorithm has been shown to be robust, even with a high ratio of missing data (Khan et al. 2018, 2019; Schomaker and Heumann 2018).

This study addressed four research questions. First, the stability of performance in the preferential looking and mismatch paradigm were examined across assessment points. Second, associations between these two paradigms were investigated using zero‐order correlations. Third, concurrent and later associations between the two eye tracking paradigms and parental reports of word comprehension and production (based on SE‐CDI) at 10, 11.5, 18 and 24 months were assessed through zero‐order correlations. Fourth, partial correlations were conducted to control for potential effects of sociodemographic variables on these associations. As the main analyses demonstrated both expected and unexpected correlations between the eye‐tracking paradigms and the SE‐CDI, additional exploratory analyses were conducted to better understand the eye‐tracking measures, including evaluation of children's attentiveness to the stimuli in the eye‐tracking tasks, group level performance, and exposure to the words used in the eye‐tracking paradigms.

## 3 Results

### 3.1 Descriptive Statistics, Missing Data, Outcome Measure Distribution, and Sensitivity Analysis

First, we examined the amount of missing and excluded data in our primary outcome variables. See Supporting Information S1: Table S1 for details. We used the EM algorithm to impute the missing data. Little's MCAR test indicated that data were missing completely at random (*p* = 0.29), which supports the use of EM imputation appropriate (Baraldi and Enders 2010). We note that the EM algorithm can produce negative and positive values beyond the observed range (see e.g., Baraldi and Enders 2010; Sammaknejad et al. 2019); for instance, some estimated SE‐CDI values were negative. After imputation, we examined the median, spread, and distributions of the primary outcome variables for the full sample (*N* = 70), see Table 3 and Supporting Information S1: Figures S2–S7. A supplementary analysis compared the non‐imputed data with the imputed data and revealed generally similar patterns, lending credibility to the findings (see Supporting Information S1: Tables S3 and S4 and Figures S8–S21).

**TABLE 3:** Descriptive data showing median, standard deviation (SD), skewness and kurtosis for all primary outcome measures.

Task	Median	SD	Skewness	Kurtosis
SE‐CDI				
Word comprehension				
10 months	21.6	16.01	0.55	0.17
11.5 months	38	19.99	0.29	−0.40
18 months	71.2	12.54	−1.36	2.90
24 months	86	5.82	−1.66	5.78
Word production				
10 months	1	3.95	1.66	2.99
11.5 months	3	6.33	1.34	2.02
18 months	20.5	21.24	0.44	1.38
24 months	75.5	19.86	−1.36	1.65
Preferential looking paradigm				
Proportion looking time				
10 months	0.05	0.12	0.30	0.20
11.5 months	0.02	0.10	−0.07	1.89
18 months	0.11	0.15	−0.16	−0.07
Mismatch paradigm				
Pupil size difference score (mm)				
10 months	−0.02	0.13	0.42	1.65
11.5 months	0.01	0.12	0.85	0.35

Abbreviation: SE‐CDI = A Swedish short form of the early vocabulary checklist.

A post hoc sensitivity analysis in G*Power (Faul et al. 2007) indicated that with *N* = 70, *α* = 0.05, two‐tailed, and 80% power, the study can detect a minimum correlation of *r* = 0.32—that is, the study is adequately powered to detect moderate‐sized correlations but may not capture smaller effects reliably.

### 3.2 Stability of Performance Across Time in the Preferential Looking and Mismatch Paradigms

The first research question investigated whether children's performance on the eye‐tracking paradigms remain stable over time. For the preferential looking paradigm, there was a significant correlation between 10‐ and 18‐month performance (rho *=* 0.31, *p* = 0.009), indicating stability over that 8‐month period. For both paradigms, there were there were no significant correlations between 10‐ and 11.5‐months performance. See Table 4 for the full correlation matrix for the eye‐tracking outcome measures.

**TABLE 4:** Correlations between the SE‐CDI and performance on the preferential looking and mismatch paradigms at 10, 11.5, 18 and 24 months.

Variable	1	2	3	4	5	6	7	8	9	10	11	12	13
1. Com 10 months	—	**0.57** **	**0.85** **	**0.50** **	**0.56** **	**0.45** **	**0.47** **	0.11	−0.18	0.19	−0.27*	0.19	−0.25*
2. Pro 10 months		—	**0.59** **	**0.78** **	**0.54** **	**0.58** **	**0.43** **	0.25*	−0.09	0.15	−0.05	−0.03	−0.08
3. Com 11.5 months			—	**0.55** **	**0.67** **	**0.48** **	**0.43** **	0.09	−0.10	0.20	−0.16	0.12	−**0.30** *
4. Pro 11.5 months				—	**0.40** **	**0.41** **	0.33*	0.11	0.03	0.10	−**0.28** *	0.19	0.01
5. Com 18 months					—	**0.72** **	**0.76** **	**0.33** **	0.04	0.21	0.04	−0.11	−**0.40** **
6. Pro 18 months						—	**0.72** **	**0.52** **	0.04	0.12	**0.32** **	−0.08	−**0.30** *
7. Com 24 months							—	**0.64** **	0.05	0.14	0.15	−0.25*	−**0.38** **
8. Pro 24 months								—	0.06	0.03	**0.30** *	−0.19	−**0.55** **
9. PLP 10 months									—	−0.19	**0.31** **	0.24*	0.18
10. PLP 11.5 months										—	−0.01	−0.01	0.06
11. PLP 18 months											—	**−0.30** *	0.06
12. MM 10 months												—	−0.05
13. MM 11.5 months													—

*Note:* This table contains Spearman correlation coefficients. Bold indicates that the correlation held after controlling for socio‐demographic variables.

Abbreviations: Com = SE‐CDI word comprehension sub‐scale, MM = Mismatch paradigm, PLP = preferential looking paradigm, Pro = SE‐CDI word production sub‐scale, SE‐CDI = Swedish Early Communicative Development Inventories.

* *p* < 0.05.

** *p* < 0.01.

***p < 0.001.

#### 3.2.1 Correlations Between Performance in the Preferential Looking and Mismatch Paradigms

The second aim was to determine whether performance in the two eye‐tracking paradigms was related. Results showed that mismatch performance at 10 months correlated with preferential looking performance at 10 (rho = 0.24, *p* = 0.04) and 18 months (rho *=* −0.30, *p* = 0.01).

The change in correlation direction over time suggests that the relationship between the two paradigms varies over age.

### 3.3 Correlations Between Preferential Looking and Mismatch Paradigm Performance and Vocabulary Development as Measured by Parental Reports

The third aim was to examine whether the children's eye‐tracking performance correlated with parental reports of vocabulary. We note that most SE‐CDI vocabulary measures were significantly intercorrelated (see Table 4). For the preferential looking paradigm, performance at 18 months was negatively correlated with word comprehension at 10 months (rho = −0.27, *p* = 0.023), and word production at 12 months (rho = −0.28, *p* = 0.019), but positively correlated with word production at 18 months (rho = 0.32, *p* = 0.006) and 24 months (rho *=* 0.30, *p* = 0.013). No other significant correlations emerged.

Regarding the mismatch paradigm, performance at 10 months was negatively correlated with word comprehension at 24 months (rho = −0.25, *p* = 0.034). At 11.5 months of age, performance was negatively correlated with word comprehension at 10 months (rho = −0.25, *p* = 0.04), 12 months (rho = −0.30, *p* = 0.012), 18 months (rho = −0.39, *p* < 0.001), and 24 months (rho = −0.38, *p* = 0.001). Negative correlations were also found with word production at 18 months (rho = −0.30, *p* = 0.011) and 24 months (rho = −0.55, *p* < 0.001). We found no other significant correlations. This pattern of correlations showed that increased pupil dilation on congruent rather than incongruent trials was linked to larger vocabulary size. Scatterplots in the Supporting Information S1 illustrate these correlations.

### 3.4 Changes in Observed Correlations When Controlling for Socio‐Demographic Variables

The fourth aim focused on whether the observed associations between the eye‐tracking paradigms and SE‐CDI persisted after controlling for sociodemographic data. Partial correlations were conducted to account for these variables (see Supporting Information S1: Table S5 for the entire matrix). For the preferential looking paradigm, performance at 10 months was no longer significantly correlated to mismatch at 10 months (rho = 0.25, *p* = 0.07). Similarly, the correlation between 18‐month performance and SE‐CDI word comprehension at 10 months was no longer significant (rho *=* −0.24, *p* = 0.087).

In the mismatch paradigm, the correlation between 10‐month performance and SE‐CDI word comprehension at 24 months was no longer significant (rho = −0.15, *p* = 0.29). Likewise, the correlation between 11.5‐month performance and SE‐CDI word comprehension at 10 months was no longer significant (rho = −0.25, *p* = 0.07).

For all other associations, the correlations held, and the directions of the correlations remained the same. Table 4 highlights the correlation coefficients in bold that remained significant after adjusting for sociodemographic variables.

### 3.5 Exploratory Analysis of Performance in the Preferential Looking and Mismatch Paradigms

Following the main analyses, exploratory investigations were conducted to understand the absence of correlations involving the preferential looking paradigm at 11.5 months, the lack of correlation between the mismatched paradigm at 10 and 11.5 months, and the strong correlations between the mismatched paradigm at 11.5 months and SE‐CDI across several assessment points.

For the preferential looking paradigm at 11.5 months, an examination of total looking (target plus distractor) indicated that children spent 1.1 s looking at 10 months, 1.3 s at 11.5 months, and 1.6 s at 18 months. Paired *t‐*tests demonstrated significant differences in total looking time across all time‐points (*t*s = −7.5 to 2.5; *p*s < 0.05), suggesting that attentiveness to the stimuli increased with age.

For the mismatch paradigm at 10 and 11.5 months, a paired *t*‐test showed no significant difference between looking time between these ages, *t*(28) = −1.7, *p* = 0.11. However, mean performance analyses indicated that pupil dilation to incongruent trials increased at 11.5 months compared to 10 months, and a paired *t*‐test confirmed that this mean difference was significant (see Supporting Information S1).

Finally, potential effects of word exposure were examined by correlating parental reports of familiarity with the words used in the two paradigms and task performance; no significant associations emerged. Further details on these exploratory analyses can be found in Supporting Information S1.

## 4 Discussion

This study investigated the stability of early word recognition assessed through eye‐tracking paradigms, and their associations with concurrent and subsequent word comprehension and production measured by parental reports. The results indicated that performance on the preferential looking paradigm was stable over time, whereas the mismatch paradigm showed no such stability. Correlations between the two paradigms were weak, but both paradigms were associated with concurrent and later word recognition and production, with most correlations remaining significant after controlling for sociodemographic variables. The discussion begins by addressing each paradigm separately before addressing their combined implications and the study's limitations.

### 4.1 Preferential Looking Paradigm

We found partial support for stability in performance on the preferential looking paradigm. Specifically, performance at 10 months correlated positively with performance at 18 months, indicating continuity in early word recognition from infancy to toddlerhood. These findings align with Lany et al. (2018), who observed both concurrent correlations between word recognition and word comprehension at 12 months and correlations with word production at 15 months (based on CDI percentile scores). Additionally, supplemental analysis showed that children in this study performed above chance at matching spoken words to the correct referent across all ages tested, replicating Bergelson and Swingley (2012, 2015) work that showed recognition of familiar words in children between 6 and 15 months.

Interestingly, performance at 11.5 months did not correlate with performance at 10 or 18 months, indicating potential instability in this measure by the end of the first year of life. Exploratory analyses suggested increased visual exploration of stimuli at 11.5 months, which may have added noise to the measure, although the reasons for this heightened exploration are unclear.

Performance on the preferential looking paradigm also revealed age‐dependent associations with vocabulary measures. At 10 and 11.5 months, no correlations emerged with any SE‐CDI subscales, while at 18 months, performance correlated negatively with word production at 11.5 months and positively with word production at 18 and 24‐months, even after controlling for socio‐demographic variables. One explanation is that performance in infancy may be too variable to capture reliable individual differences. Whereas by toddlerhood, the task may better capture meaningful variance in early word recognition abilities.

An alternative interpretation is that there is a stable underlying word recognition ability from infancy to toddlerhood, but the relationship between gaze‐based word recognition and parental vocabulary reports shifts with age. Before the onset of spoken language, parental reports contain more errors and biases (Feldman et al. 2005, 2000), potentially leading to underestimates of infants' vocabulary (Bergelson and Swingley 2012; Golinkoff et al. 2013). As children grow older and exhibit more observable language skills, parental estimates may become more accurate, thus aligning more closely with eye‐tracking measures.

Another possibility is that methodological differences contribute to those patterns. The preferential looking paradigm measures word recognition proportionally (i.e., relative looking time), whereas the SE‐CDI uses dichotomous parent ratings for word comprehension. The continuous nature of looking time data may capture more nuanced variations in word recognition than the binary parental reports. Future research comparing preferential looking measures with other observational methods could help clarify whether the observed patterns are specific to SE‐CDI data or reflect broader trends (Bornstein and Putnick 2012).

Performance at 18 months was positively correlated with word production at 18 and 24 months, but not with word comprehension. This difference is likely due to greater variability in the word production subscale at these ages. Indeed, by 18 or 24 months, most children are estimated to understand most words the SE‐CDI short form, while it still differs greatly whether they say the words or not (see Table 4).

### 4.2 Mismatch Paradigm

Performance in the mismatch paradigm was not stable over time. At 10 months, infants exhibited greater pupil dilation in response to matching stimuli (congruent trials), whereas at 11.5 months, they showed greater pupil dilation to mismatching stimuli (incongruent trials; see Supporting Information S1). This shift suggests developmental changes in how infants respond to match versus mismatch conditions, possibly due to expanded vocabulary at 11.5 months driving a stronger reaction to mismatching stimuli. Future research could add another time‐point (e.g., 18 months), to see if this pattern persists over a larger timespan. Notably, while Parise and Csibra (2012) found a N400‐like response in infants as young as 9 months, Friedrich and Friederici (2005),  (2008) observed reliable mismatch responses only at 14 months of age, suggesting that infants younger than 14 months may detect mismatching inconsistently at a group level.

Unexpectedly, mismatch performance at 10 months did not correlate with any SE‐CDI measure, while performance at 11.5 months was negatively correlated with both concurrent and later vocabulary. This finding supports the notion that performance in the mismatch paradigm changes with age and it also suggests that pupil dilation in matching (rather than mismatching) stimuli is related to vocabulary development at 11.5 months. A speculative explanation is that infants at this age are particularly attuned to consistent label‐object pairings in everyday life, focusing on how words and objects match as part of fundamental word‐learning (Saxton 2010). By 14 months or older infants may then shift to detecting and responding more systematically to mismatches (Friedrich and Friederici 2005, 2008). However, more research is needed before we can make any strong claims.

Although, the mismatch paradigm is designed to measure infants' ability to detect mismatches between labels and objects (Parise and Csibra 2012), the present result suggest that pupil dilation may reflect a different underlying mechanism, such as arousal (Kloosterman et al. 2015). Moreover, the lack of consistent correlations between mismatch and preferential looking performance complicates our understanding of what the mismatch paradigm measures. Comparing pupil dilation data with ERP data in the same sample might clarify the mechanisms behind these pupillary responses, though such an investigation falls outside the scope of this study.

### 4.3 Combined Implications of the Two Paradigms

Taken together, the preferential looking and mismatch paradigms both provide insights into early word recognition and its link to vocabulary development. Their performance measures showed associations with concurrent and later vocabulary, highlighting the role for early word recognition in facilitating both word comprehension and production. These findings also suggest that eye‐tracking measures may serve as useful complement to parental report in infancy and toddlerhood. However, further research is needed to clarify the developmental trajectories and mechanistic underpinnings of each paradigm better before offering strong conclusions or recommendations.

### 4.4 Strengths and Limitations

A key concern involves the amount of missing data in the eye‐tracking‐based paradigms. In the mismatch paradigm, approximately 60% of the data from the first assessment and 50% from the second assessment were initially deemed valid multiple imputation with the EM algorithm (see Table S1). Some of these missing data stem from technical issues with the eye‐tracker, resulting in the loss of 12 participants' data at both 10 and at 11.5 months. While our attrition rate resembles that of Parise and Csibra (2012), who reported valid data for about 57% of their sample, the pupil‐dilation outcome measure is indeed more vulnerable to noise and artifacts (e.g., blinks, eye‐ and head‐movements) than the looking‐time measure used in the preferential looking paradigm.

Despite these challenges, the study's longitudinal design with multiple assessments improves on previous research that has often relied on single time points or cross‐sectional design. By tracking the same infants over time, we offer clearer insights into how preferential looking and mismatch performances relate to concurrent and later vocabulary, and their stability across infancy and toddlerhood. These findings enhance our understanding of eye‐tracking methods in language development and emphasize the need for further refinement of these paradigms, especially for younger infants.

## 5 Conclusion

In sum, the preferential looking paradigm appeared to capture stable individual differences in early word recognition from infancy into toddlerhood and showed meaningful links with both word comprehension and production. These results suggest that the preferential looking paradigm may serve as a valuable complement to parental reports, offering an objective assessment of word recognition. Nonetheless, further research is needed to elucidate early within‐child variability and to refine the paradigm for broader application.

By contrast, the mismatch paradigm revealed a possible developmental shift in how infants respond to the task, likely contributing to the lack of performance stability between 10 and 11.5 months. While mismatch performance was associated with both concurrent and later word comprehension and production, the unexpected direction of these correlations raises interesting questions about the underlying cognitive or attentional mechanism. These findings highlight the need for further research to clarify what the mismatch paradigm measures at different developmental stages and to evaluate its utility in assessing early word recognition.

## Author Contributions

**Anton Gerbrand:** conceptualization, data curation, formal analysis, methodology, visualization, writing – original draft, writing – review and editing. **Johan Wengman:** data curation, investigation, project administration, writing – review and editing. **Linda Forssman:** conceptualization, data curation, formal analysis, funding acquisition, methodology, project administration, resources, supervision, visualization, writing – original draft, writing – review and editing.

## Ethics Statement

The current study was approved by the Swedish Ethical Review Authority (Etikprövningsmyndigheten, reference number 2019‐03140).

## Conflicts of Interest

The authors declare no conflicts of interest.

## Supporting information

Supporting Information S1

## Data Availability

All data, analyses, material necessary for replicating the findings can be found at: https://osf.io/sbzrt/?view_only=b6d2cd11ade848ef80a39f438738795c. Analyses were also pre‐registered, which can be found at: https://osf.io/4qekb/?view_only=7e303836d2d4480eab2f42fdea8e57f6.

## References

[infa70028-bib-0001] Axfors, C. , E. Bränn , H. E. Henriksson , et al. 2019. “Cohort Profile: The Biology, Affect, Stress, Imaging and Cognition (BASIC) Study on Perinatal Depression in a Population‐Based Swedish Cohort.” BMJ Open 9, no. 10: e031514. 10.1136/bmjopen-2019-031514. PMC6830667 31641004

[infa70028-bib-0002] Baraldi, A. N. , and C. K. Enders . 2010. “An Introduction to Modern Missing Data Analyses.” Journal of School Psychology 48, no. 1: 5–37. 10.1016/j.jsp.2009.10.001. 20006986

[infa70028-bib-0003] Bayley, N. 2009. Bayley‐III: Bayley Scales of Infant and Toddler Development. Giunti OS.

[infa70028-bib-0004] Bergelson, E. 2020. “The Comprehension Boost in Early Word Learning: Older Infants Are Better Learners.” Child Development Perspectives 14, no. 3: 142–149. 10.1111/cdep.12373. 33569084 PMC7872330

[infa70028-bib-0005] Bergelson, E. , and D. Swingley . 2012. “At 6–9 Months, Human Infants Know the Meanings of Many Common Nouns.” Proceedings of the National Academy of Sciences 109, no. 9: 3253–3258. 10.1073/pnas.1113380109. PMC3295309 22331874

[infa70028-bib-0006] Bergelson, E. , and D. Swingley . 2015. “Early Word Comprehension in Infants: Replication and Extension.” Language Learning and Development 11, no. 4: 369–380. 10.1080/15475441.2014.979387. 26664329 PMC4671511

[infa70028-bib-0007] Bishop, D. V. , G. Holt , E. Line , D. McDonald , S. McDonald , and H. Watt . 2012. “Parental Phonological Memory Contributes to Prediction of Outcome of Late Talkers From 20 Months to 4 Years: A Longitudinal Study of Precursors of Specific Language Impairment.” Journal of Neurodevelopmental Disorders 4: 1–12. 10.1186/1866-1955-4-3. 22958373 PMC3374292

[infa70028-bib-0008] Bornstein, M. H. , and D. L. Putnick . 2012. “Stability of Language in Childhood: A Multiage, Multidomain, Multimeasure, and Multisource Study.” Developmental Psychology 48, no. 2: 477–491. 10.1037/a0025889. 22004343 PMC3412562

[infa70028-bib-0009] De Houwer, A. , M. H. Bornstein , and S. De Coster . 2006. “Early Understanding of Two Words for the Same Thing: A CDI Study of Lexical Comprehension in Infant Bilinguals.” International Journal of Bilingualism 10, no. 3: 331–347. 10.1177/13670069060100030401.

[infa70028-bib-0010] Dong, Y. , S. X. Y. Wu , W. Y. Dong , and Y. Tang . 2020. “The Effects of Home Literacy Environment on Children's Reading Comprehension Development: A Meta‐Analysis.” Educational Sciences: Theory and Practice 20, no. 2: 63–82. 10.12738/jestp.2020.2.005.

[infa70028-bib-0011] Donnelly, S. , and E. Kidd . 2020. “Individual Differences in Lexical Processing Efficiency and Vocabulary in Toddlers: A Longitudinal Investigation.” Journal of Experimental Child Psychology 192: 104781. 10.1016/j.jecp.2019.104781. 31981753

[infa70028-bib-0012] D'Souza, D. , H. D'Souza , and A. Karmiloff‐Smith . 2017. “Precursors to Language Development in Typically and Atypically Developing Infants and Toddlers: The Importance of Embracing Complexity.” Journal of Child Language 44, no. 3: 591–627. 10.1017/s030500091700006x. 28393740

[infa70028-bib-0013] Eriksson, M. , M. Westerlund , and E. Berglund . 2002. “A Screening Version of the Swedish Communicative Development Inventories Designed for Use with 18‐Month‐Old Children.” Journal of Speech, Language, and Hearing Research: Journal of Speech Language Hearing Research 45, no. 5: 948–960. 10.1044/1092-4388(2002/077. 12381052

[infa70028-bib-0014] Faul, F. , E. Erdfelder , A. G. Lang , and A. Buchner . 2007. “G* Power 3: A Flexible Statistical Power Analysis Program for the Social, Behavioral, and Biomedical Sciences.” Behavior Research Methods 39, no. 2: 175–191. 10.3758/bf03193146. 17695343

[infa70028-bib-0015] Feldman, H. M. , P. S. Dale , T. F. Campbell , et al. 2005. “Concurrent and Predictive Validity of Parent Reports of Child Language at Ages 2 and 3 Years.” Child Development 76, no. 4: 856–868. 10.1111/j.1467-8624.2005.00882.x. 16026501 PMC1350485

[infa70028-bib-0016] Feldman, H. M. , C. A. Dollaghan , T. F. Campbell , M. Kurs‐Lasky , J. E. Janosky , and J. L. Paradise . 2000. “Measurement Properties of the MacArthur Communicative Development Inventories at Ages One and Two Years.” Child Development 71, no. 2: 310–322. 10.1111/1467-8624.00146. 10834466

[infa70028-bib-0017] Fenson, L. , S. Pethick , C. Renda , J. L. Cox , P. S. Dale , and J. S. Reznick . 2000. “Short‐Form Versions of the MacArthur Communicative Development Inventories.” Applied Psycholinguistics 21, no. 1: 95–116. 10.1017/s0142716400001053.

[infa70028-bib-0018] Fernald, A. , and V. A. Marchman . 2012. “Individual Differences in Lexical Processing at 18 Months Predict Vocabulary Growth in Typically Developing and Late‐Talking Toddlers.” Child Development 83, no. 1: 203–222. 10.1111/j.1467-8624.2011.01692.x. 22172209 PMC3266972

[infa70028-bib-0019] Fernald, A. , R. Zangl , A. L. Portillo , and V. A. Marchman . 2008. “Looking While Listening: Using Eye Movements to Monitor Spoken Language.” Developmental Psycholinguistics: On‐Line Methods in Children’s Language Processing 44: 97.

[infa70028-bib-0020] Forssman, L. 2022. REaL. Databrary. https://nyu.databrary.org/volume/1506.

[infa70028-bib-0021] Forssman, L. , and J. M. Gottwald . 2022. “The Impact of Interactive Book Sharing on Child Cognitive and Socio‐Cognitive Development (The REaL Trial): Study Protocol for a Randomized Controlled Trial.” Trials 23, no. 1: 802. 10.1186/s13063-022-06733-8. 36153547 PMC9509634

[infa70028-bib-0022] Friedrich, M. , and A. D. Friederici . 2005. “Phonotactic Knowledge and Lexical‐Semantic Processing in One‐Year‐Olds: Brain Responses to Words and Nonsense Words in Picture Contexts.” Journal of Cognitive Neuroscience 17, no. 11: 1785–1802. 10.1162/089892905774589172. 16269114

[infa70028-bib-0023] Friedrich, M. , and A. D. Friederici . 2008. “Neurophysiological Correlates of Online Word Learning in 14‐Month‐Old Infants.” NeuroReport 19, no. 18: 1757–1761. 10.1097/wnr.0b013e328318f014. 18955904

[infa70028-bib-0024] Golinkoff, R. M. , W. Ma , L. Song , and K. Hirsh‐Pasek . 2013. “Twenty‐Five Years Using the Intermodal Preferential Looking Paradigm to Study Language Acquisition: What Have We Learned?” Perspectives on Psychological Science 8, no. 3: 316–339. 10.1177/1745691613484936. 26172975

[infa70028-bib-0025] Hepach, R. , and G. Westermann . 2013. “Infants’ Sensitivity to the Congruence of Others’ Emotions and Actions.” Journal of Experimental Child Psychology 115, no. 1: 16–29. 10.1016/j.jecp.2012.12.013. 23454359

[infa70028-bib-0026] Hoff, E. 2013. “Interpreting the Early Language Trajectories of Children From Low‐SES and Language Minority Homes: Implications for Closing Achievement Gaps.” Developmental Psychology 49, no. 1: 4–14. 10.1037/a0027238. 22329382 PMC4061698

[infa70028-bib-0027] Houston‐Price, C. , E. Mather , and E. Sakkalou . 2007. “Discrepancy Between Parental Reports of Infants' Receptive Vocabulary and Infants' Behaviour in a Preferential Looking Task.” Journal of Child Language 34, no. 4: 701–724. 10.1017/s0305000907008124. 18062356

[infa70028-bib-0028] Jahn‐Samilo, J. , J. Goodman , E. Bates , and M. Sweet . 2001. Vocabulary Learning in Children From 8 to 30 Months of Age: A Comparison of Parental Report and Laboratory Measures. Manuscript Submitted for Publication.

[infa70028-bib-0029] Junge, C. , M. Boumeester , D. L. Mills , M. Paul , and S. H. Cosper . 2021. “Development of the N400 for Word Learning in the First 2 Years of Life: A Systematic Review.” Frontiers in Psychology 12: 689534. 10.3389/fpsyg.2021.689534. 34276518 PMC8277998

[infa70028-bib-0030] Khan, S. S. , A. Ahmad , and A. Mihailidis . 2018. “Bootstrapping and Multiple Imputation Ensemble Approaches for Missing Data.” arXiv preprint arXiv:1802.00154, 1–14.

[infa70028-bib-0031] Khan, S. S. , A. Ahmad , and A. Mihailidis . 2019. “Bootstrapping and Multiple Imputation Ensemble Approaches for Classification Problems.” Journal of Intelligent and Fuzzy Systems 37, no. 6: 7769–7783. 10.3233/jifs-182656.

[infa70028-bib-0032] Kloosterman, N. A. , T. Meindertsma , A. M. van Loon , V. A. Lamme , Y. S. Bonneh , and T. H. Donner . 2015. “Pupil Size Tracks Perceptual Content and Surprise.” European Journal of Neuroscience 41, no. 8: 1068–1078. 10.1111/ejn.12859. 25754528

[infa70028-bib-0033] Kuipers, J. R. , and G. Thierry . 2011. “N400 Amplitude Reduction Correlates With an Increase in Pupil Size.” Frontiers in Human Neuroscience 5: 61. 10.3389/fnhum.2011.00061. 21747766 PMC3128247

[infa70028-bib-0034] Lany, J. , M. Giglio , and M. Oswald . 2018. “Infants’ Lexical Processing Efficiency Is Related to Vocabulary Size by One Year of Age.” Infancy 23, no. 3: 342–366. 10.1111/infa.12228.

[infa70028-bib-0035] Leppänen, J. M. , L. Forssman , J. Kaatiala , S. Yrttiaho , and S. Wass . 2015. “Widely Applicable MATLAB Routines for Automated Analysis of Saccadic Reaction Times.” Behavior Research Methods 47, no. 2: 538–548. 10.3758/s13428-014-0473-z. 24788324 PMC4427653

[infa70028-bib-0036] Marchman, V. A. , K. A. Adams , E. C. Loi , A. Fernald , and H. M. Feldman . 2016. “Early Language Processing Efficiency Predicts Later Receptive Vocabulary Outcomes in Children Born Preterm.” Child Neuropsychology 22, no. 6: 649–665. 10.1080/09297049.2015.1038987. 26031342 PMC4668235

[infa70028-bib-0037] Marchman, V. A. , and A. Fernald . 2008. “Speed of Word Recognition and Vocabulary Knowledge in Infancy Predict Cognitive and Language Outcomes in Later Childhood.” Developmental Science 11, no. 3: F9–F16. 10.1111/j.1467-7687.2007.00671.x. 18466367 PMC2905590

[infa70028-bib-0038] Meylan, S. C. , and E. Bergelson . 2022. “Learning Through Processing: Toward an Integrated Approach to Early Word Learning.” Annual review of linguistics 8, no. 1: 77–99. 10.1146/annurev-linguistics-031220-011146. PMC9037961 35481110

[infa70028-bib-0040] Nyström, P. , T. Falck‐Ytter , and G. Gredebäck . 2016. “The TimeStudio Project: An Open Source Scientific Workflow System for the Behavioral and Brain Sciences.” Behavior Research Methods 48, no. 2: 542–552. 10.3758/s13428-015-0616-x. 26170051 PMC4891379

[infa70028-bib-0041] Parise, E. , and G. Csibra . 2012. “Electrophysiological Evidence for the Understanding of Maternal Speech by 9‐Month‐Old Infants.” Psychological Science 23, no. 7: 728–733. 10.1177/0956797612438734. 22692337 PMC4641316

[infa70028-bib-0042] Peter, M. S. , S. Durrant , A. Jessop , A. Bidgood , J. M. Pine , and C. F. Rowland . 2019. “Does Speed of Processing or Vocabulary Size Predict Later Language Growth in Toddlers?” Cognitive Psychology 115: 101238. 10.1016/j.cogpsych.2019.101238. 31539813

[infa70028-bib-0043] Piot, L. , N. Havron , and A. Cristia . 2022. “Socioeconomic Status Correlates With Measures of Language Environment Analysis (LENA) System: A Meta‐Analysis.” Journal of Child Language 49, no. 5: 1037–1051. 10.1017/s0305000921000441. 34180383

[infa70028-bib-0044] Sammaknejad, N. , Y. Zhao , and B. Huang . 2019. “A Review of the Expectation Maximization Algorithm in Data‐Driven Process Identification.” Journal of Process Control 73: 123–136. 10.1016/j.jprocont.2018.12.010.

[infa70028-bib-0039] Sander‐Montant, A. , M. L. Pérez , and K. Byers‐Heinlein . 2023. “The More They Hear the More They Learn? Using Data From Bilinguals to Test Models of Early Lexical Development.” Cognition 238, 105525. 37402336 10.1016/j.cognition.2023.105525

[infa70028-bib-0045] Saxton, M. 2010. Child Language: Acquisition and Development. SAGE Publications.

[infa70028-bib-0046] Schomaker, M. , and C. Heumann . 2018. “Bootstrap Inference When Using Multiple Imputation.” Statistics in Medicine 37, no. 14: 2252–2266. 10.1002/sim.7654. 29682776 PMC5986623

[infa70028-bib-0047] Swingley, D. 2009. “Onsets and Codas in 1.5‐Year‐Olds’ Word Recognition.” Journal of Memory and Language 60, no. 2: 252–269. 10.1016/j.jml.2008.11.003. 20126290 PMC2678748

[infa70028-bib-0048] Tincoff, R. , and P. W. Jusczyk . 1999. “Some Beginnings of Word Comprehension in 6‐Month‐Olds.” Psychological Science 10, no. 2: 172–175. 10.1111/1467-9280.00127.

[infa70028-bib-0049] Venker, C. E. , E. Haebig , J. Edwards , J. R. Saffran , and S. Ellis Weismer . 2016. “Brief Report: Early Lexical Comprehension in Young Children With ASD: Comparing Eye‐Gaze Methodology and Parent Report.” Journal of Autism and Developmental Disorders 46, no. 6: 2260–2266. 10.1007/s10803-016-2747-z. 26883646 PMC4860357

[infa70028-bib-0050] Woodward, A. L. , E. M. Markman , and C. M. Fitzsimmons . 1994. “Rapid Word Learning in 13‐and 18‐Month‐Olds.” Developmental Psychology 30, no. 4: 553–566. 10.1037//0012-1649.30.4.553.

