# Supplementary material for: Gazing Into Language Development: Exploring Individual Variability in Early Word Recognition in Infancy Through Eye‐Tracking
Source: Infancy. 2025 Jun 26;30(4):e70028. doi: 10.1111/infa.70028 (PMC12199785; doi:10.1111/infa.70028)
Supplement: Supplementary file 1 — Supporting Information S1 [file INFA-30-0-s001.docx]

**Supplementary Information:**

**Gazing into language development: Exploring individual variability in early word recognition in infancy through eye-tracking**

**Missing Data on Primary Outcome Measures**

Table S1 presents the percentage of missing data for primary outcome measures based on the initial sample size (n = 70). Missing data includes cases where outcome measures did not meet inclusion criteria, such as low-quality data or technical issues, participants not attending lab assessments, or parents providing incomplete questionnaire responses.

**Table S1.**

*Percentage of missing data for language outcome measures for each time point based on initial sample size (n = 70).*

| **Language measures** | | | |
| --- | --- | --- | --- |
| **Timepoint** | **SE-SE-CDI** | **Preferential Looking** | **Mismatch Paradigm** |
| 10 months | 7.1% | 31.4% | 38.6% |
| 11.5 months | 20% | 20% | 47.1% |
| 18 months | 15.7% | 25.7% |  |
| 24 months | 30% |  |  |

*Note.* SE-SE-CDI = The Swedish Early Communication Development Inventory

**Words and Sentences in Preferential Looking and Mismatch Paradigms**

Table S2 lists target words in the Preferential Looking and Mismatch Paradigms. The selected words largely overlap with previous studies (Bergelson & Swingley, 2012; Parise & Csibra 2012). All but four words also appear in the short form of the SE-SE-CDI.

| **Table S2.**  *Words and Spoken Sentences in the Preferential Looking and Mismatch Paradigms* | | | |
| --- | --- | --- | --- |
| **Preferential Looking Paradigm** | | | |
| ***Warm-up phase*** | | | |
| **Image** | **Sentence** | **Image** | **Sentence** |
| Sheep | "Can you find the sheep?" | Monkey | "Do you see the monkey?" |
| Pig | "Where's the pig?” | Frog | "Look at the frog!" |
| ***Paired-Pictures: Test Phase*** | | | |
| **Image** | **Sentence** | **Image** | **Sentence** |
| Bottle-Belly | "Where's the bottle/belly?” | Ear-Jumper | "Look at the ear/jumper!" |
| Nose-Buss | "Can you find the nose/buss?" | Eye-Spoon | "Where's the eye/spoon? |
| Foot-Swing | "Look at the foot/swing!" | Doll-Baby | "Can you find the doll/baby?" |
| Hand-Sock | "Do you see the hand/sock?" | Mouth-Pacifier | "Do you see the mouth/pacifier?" |
| **Mismatch Paradigm** | | | |
| **Image** | **Sentence** | **Image** | **Sentence** |
| Duck | “Look at the duck/teddy bear!” | Teddy Bear | “Look at the duck/teddy bear!” |
| Apple | ”Look at the apple/shoe!” | Shoe | ”Look at the apple/shoe!” |
| Cat | ”Look at the horse/cat!” | Horse | ”Look at the horse/cat!” |
| Banana | ”Look at the bird/banana!” | Bird | ”Look at the bird/banana!” |
| Hat | ”Look at the hat/ball!” | Ball | ”Look at the hat/ball!” |
| Dog | ”Look at the sandwich/dog!” | Sandwich | ”Look at the sandwich/dog!” |
| Diaper | ”Look at the lamp/diaper!” | Lamp | ”Look at the lamp/diaper!” |
| Cow | ”Look at the cow/car!” | Car | ”Look at the cow/car!” |

**Eye Tracking Pre-Processing: Areas of Interest**

In the Preferential Looking Paradigm, two Areas of Interest (AOIs) were defined: the target and the distractor. Figure S1 represents AOI definition in TimeStudio (version 3.23; Nyström et al., 2016) but does not reflect the actual stimulus presentation.

**Figure S1**

Areas of interest (AOIs) for analyzing looking time to target and distractor in the Preferential Looking Paradigm.


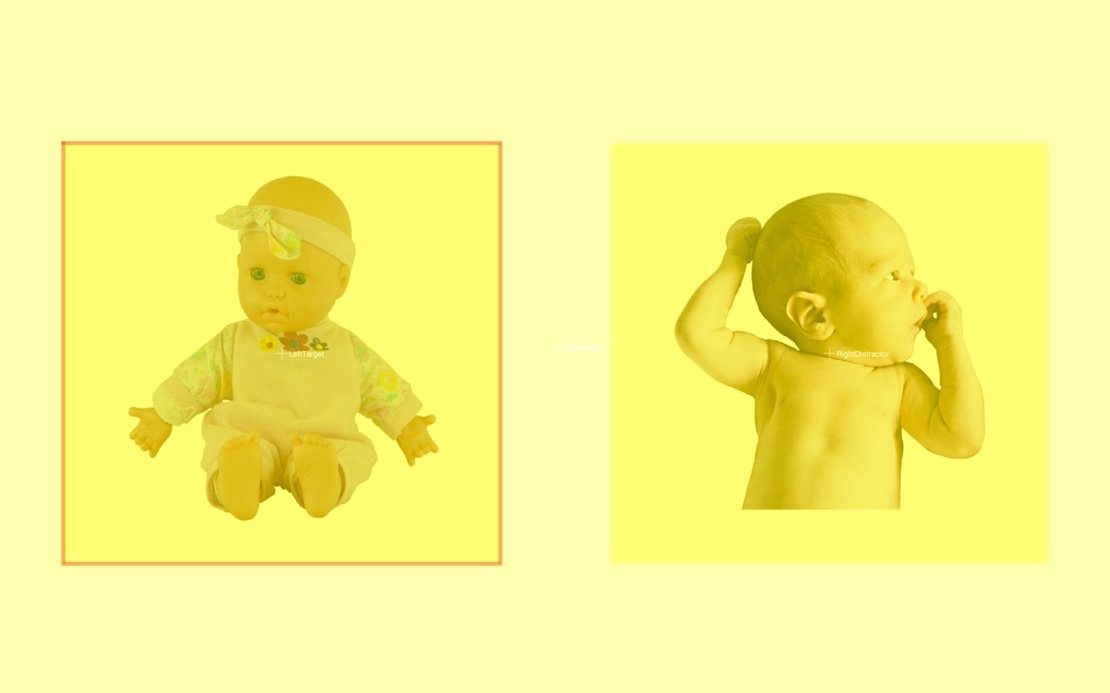


**Outcome Measure Distribution: SE-SE-CDI, Preferential Looking and Mismatched Paradigms**

**SE-SE-CDI.** We first examined the descriptive statistics and distribution of our language outcome variables, based on data imputed with EM algorithm. See the main text for the descriptive statistics. Here, we present the distribution of our data in histograms. We start by presenting the distribution of the SE-CDI, see **Figure S2 – S5**. As we can see, most SE-SE-CDI data is clearly skewed and non-normally distributed.

**Figure S2**


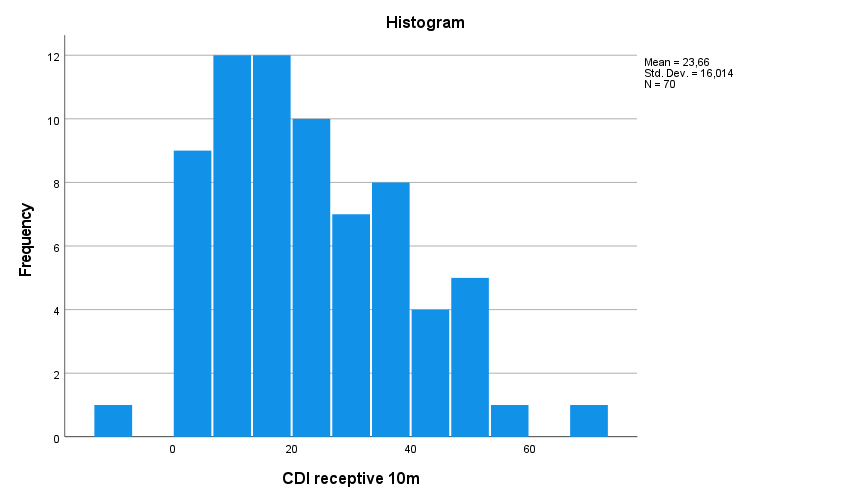

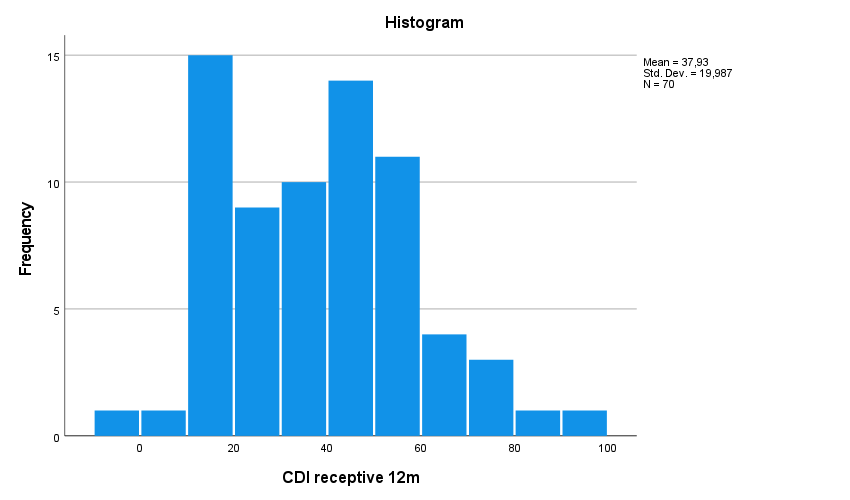


*Note.* Distribution of SE-CDI word comprehension at 10 months (left) and 11.5 months (right). The distribution at 10 months is indicative of non-normality, due to a moderate positive skewness and a negative kurtosis. The distribution at 11.5 months is indicative of normality, due to a moderate positive skew and negative kurtosis and an insignificant Shaprio-Wilk test (*p* = .18)

**Figure S3**


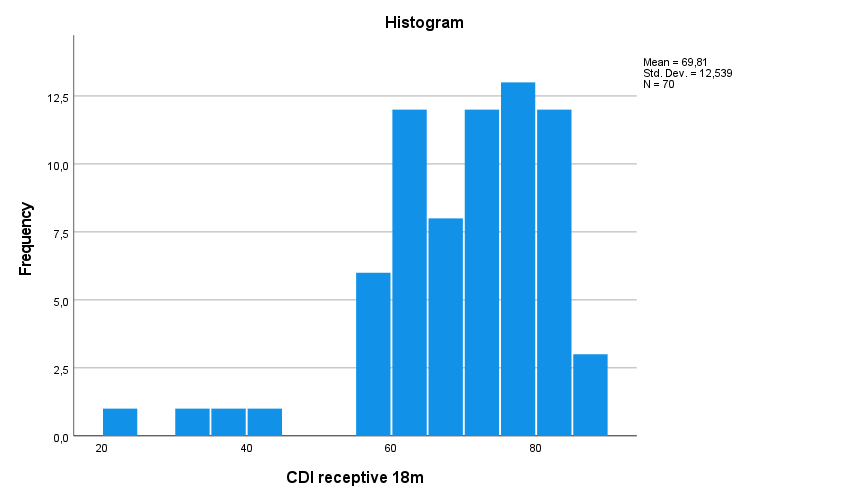

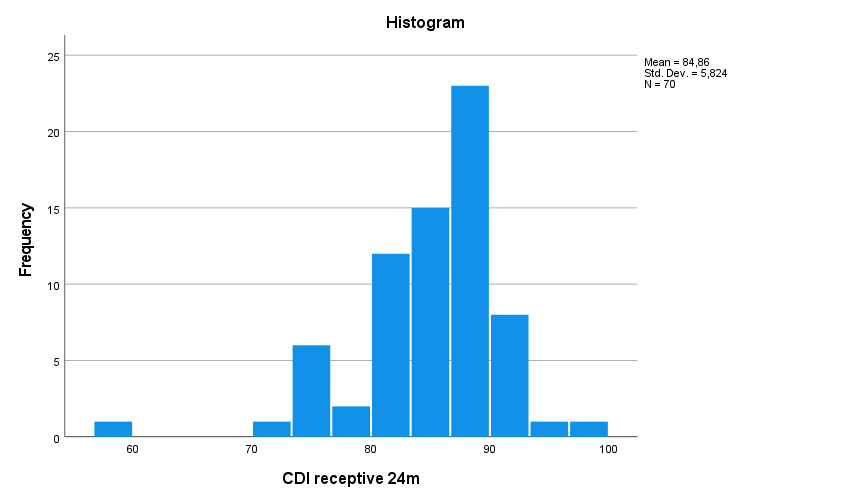


*Note.* Distribution of SE-CDI word comprehension at 18 months (left) and at 24 months (right). Both distributions are indicative of non-normality, due to a high negative skew and a negative kurtosis and a significant Shaprio-Wilk test (*p<.001*) for the data at 18 months, and due to a moderate negative skew and a positive kurtosis and a significant Shaprio- Wilk test (*p* = .02) for the data at 24 months.

**Figure S4**


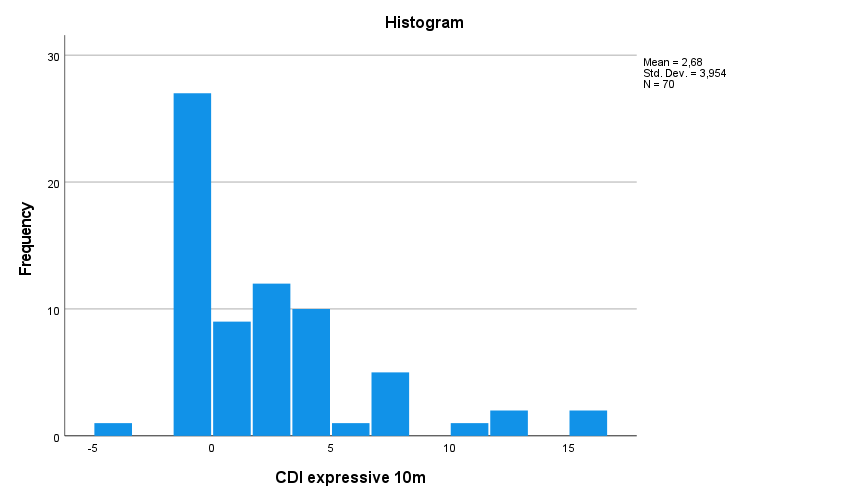

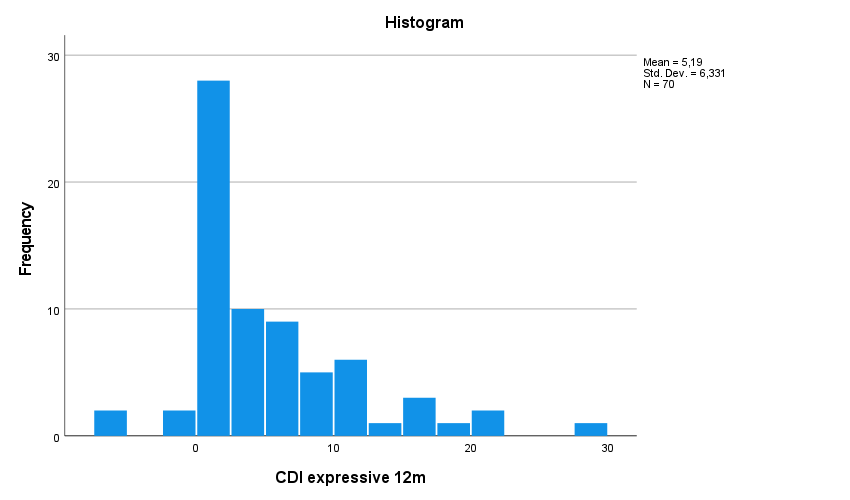


*Note.* Distribution of SE-CDI word production vocabulary at 10 months (left) and 11.5 months (right). The distributions are indicative of non-normality, due to high positive skew and significant Shaprio-Wilk tests (*p<.001)*.

**Figure S5**


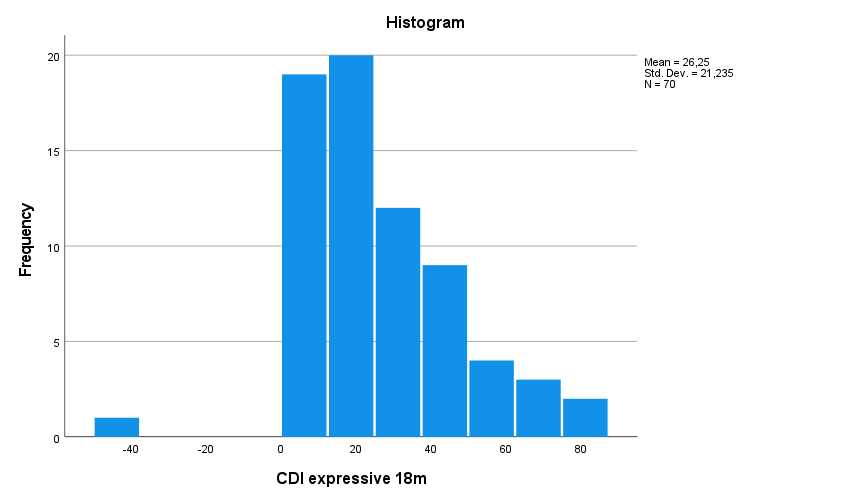

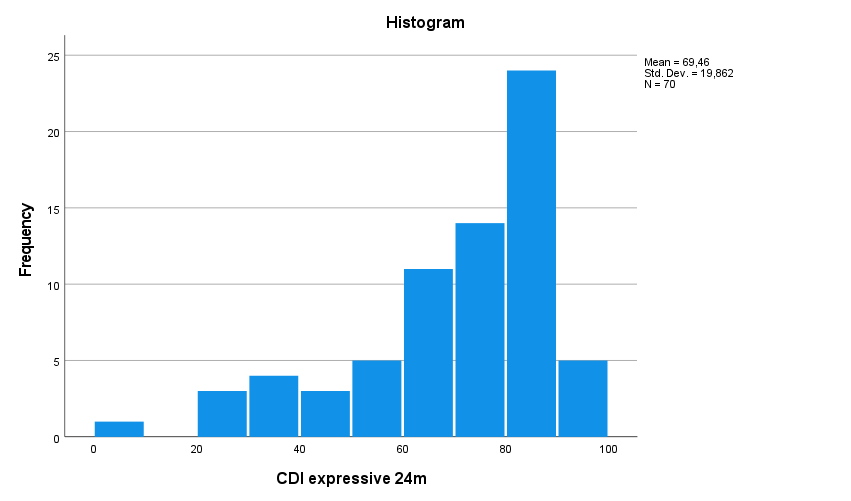


*Note.* Distribution of SE-CDI word production at 18 months (left) and 24 months (right). Both distributions are indicative of non-normality, due to a high positive skew and a negative kurtosis and a significant Shaprio-Wilk test (*p<.001)* for the data at 18 months, and due to a high negative skew and a negative kurtosis and a significant Shaprio- Wilk test (*p<.001)* for the data at 24 months.

**Preferential looking paradigm task.** Next, we examined the distribution of the preferential looking paradigm task. These distributions indicated an acceptable degree of normal distribution, see Figure S6.

**Figure S6**


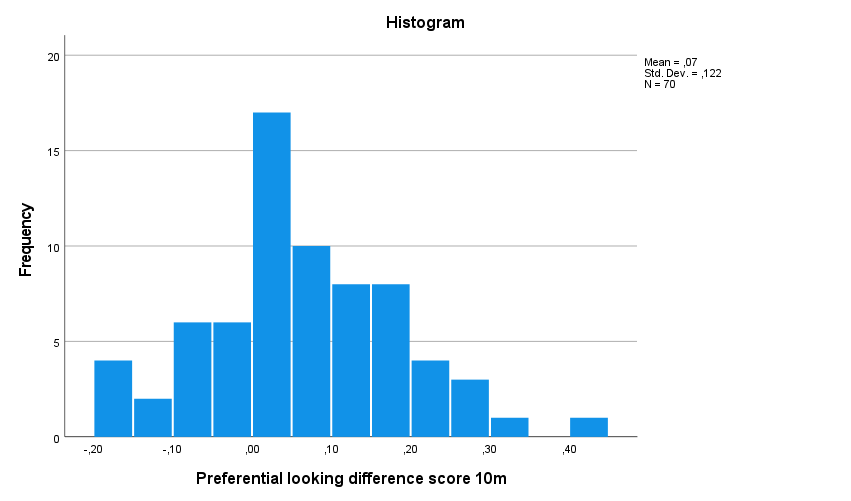

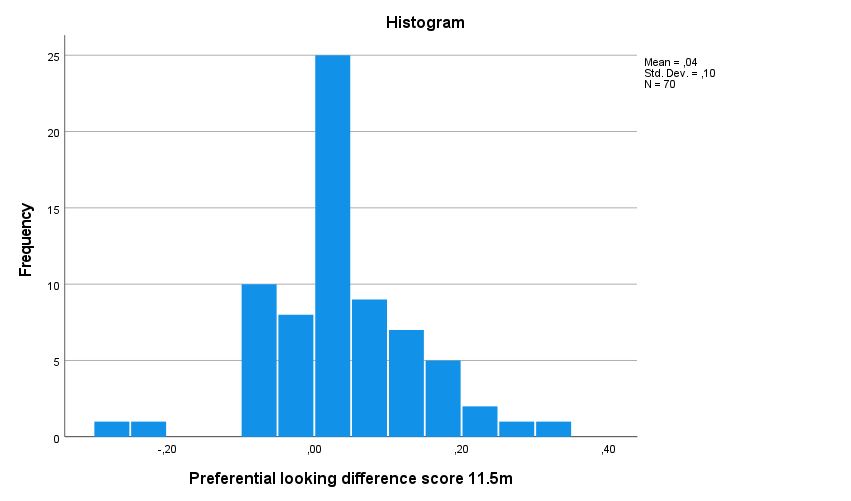

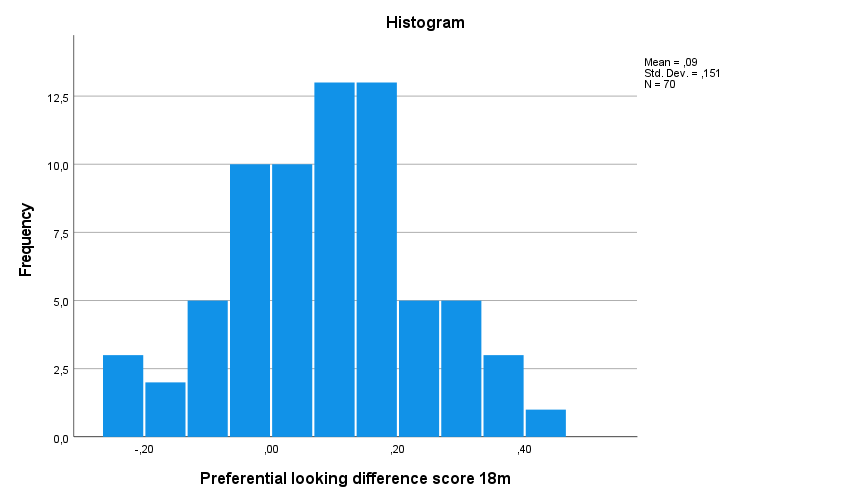


*Note.* Distribution of the preferential looking difference score at 10 months (left), 11.5 months (center) and 18 months (left). All the distribution were deemed indicative of a normality, due to low to moderate skew and insignificant Shaprio-Wilk tests (*ps* > .59), except the Shapiro-Wilk test at 11.5 months being significant (*p* = .02).

**Mismatch task.** Next, we examined the distribution of the mismatch task. These distributions did not indicate an acceptable degree of normal distribution, see Figure S7.

**Figure S7**


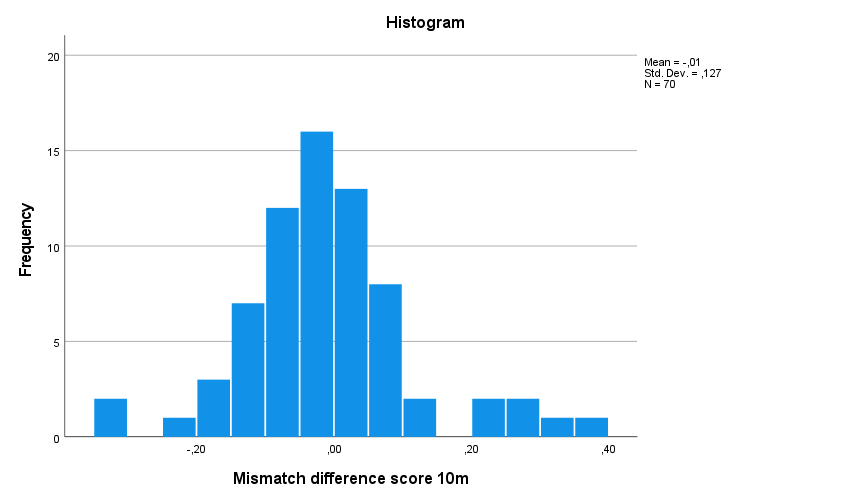

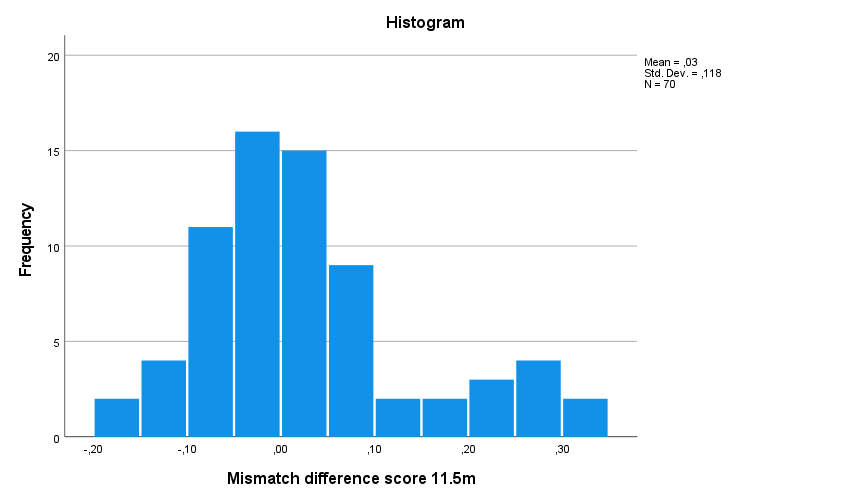


*Note.* Distribution of the mismatch difference score at 10 months (left) and 11.5 months (right). The distributions are not indicative of normality, due to a moderate to high negative skewness and a negative kurtosis and significant Shaprio-Wilk tests (*p*< .003*)*

**Exploring Group Performance, Attentiveness and Word Exposure**

**Group Performance.** In the Preferential Looking Paradigm, infants performed above chance at 10 months (M = .07, SD = .12, *t*(69) = 24.5, *p<.*001), 11.5 months (M =.04, SD = .09, *t*(69) = 3.1, *p* = .003) and 18 months (M = .09, SD = .15, *t*(69) = 5.1, *p<*.001), replicating previous studies (e.g. Bergelson and Sinwgley, 2012;2015). In the Mismatch Paradigm, pupil dilation to mismatching stimuli increased significantly between 10 (M = -.01, SD = .13) and 11.5 (M = .03, SD = .12) months, *t*(69) = -2, *p* = .045).

**Attentiveness.** In the preferential looking paradigm, participants looked significantly more towards stimuli at 11.5 months (M = 1.3s, SDs = .48) compared to 10 months (M = 1.1s, SD = .52s), *t*(61) = -2.5, *p* = .015, and looked more towards the stimuli at 18 months (M = 1.6s, SD = 2.9s) compared to at 11.5 months (*t*(52) = -5.7, *p<*.001), and compared to at 10 months (*t*(51) = -7.5, *p*<.001).

In the mismatch paradigm, paired t-test demonstrated no difference in looking time at 10 months (M = .95s, SD = .43s) and 11.5 months (M = 1.17s, SD = .37s), *t*(28) = -1.7, *p* = .11. (Note that these analyses are based on non-imputed data).

**Word Exposure.** Prior to testing, parents were asked to estimate how often their child heard the words in the task: *never (0)*, l*ess than once a week (1), a few times a week (2)*, *once per day (3)*, and *several times per day (4*), and we calculated the mean exposure of all words in each task.

For the preferential looking paradigm, mean exposure of words was 2.28 (SD = .61), and at 11.5 months the mean exposure was 2.36 (SD = .58). The estimated exposure of words did not correlate with performance at 10 months (r = -.13, *p* = .29), nor with performance at 11.5 months (r = -.16, *p* = .20). This is in line with previous research (Bergleson & Swingley, 2015).

In the mismatch paradigm, at 10 months, the mean exposure of words was 2.16 (SD= .63), and at 11.5 months the mean exposure was 2.32 (SD= .59). The estimated word exposure did not correlate with performance at 10 months (r = -.16, *p* = .19), nor with performance at 11.5 months (r = -.04, *p* = .77).

**A Comparison between Imputed Data and Original Data**

To ensure accuracy, both imputed and non-imputed data were analyzed. Imputation did not significantly alter correlation patterns. It is important to note that correlations in the non-imputed data are expected to be weaker and often non-significant due to reduced statistical power from the smaller effective sample size. **Table S3** provides descriptive statistics before and after imputation.

Looking at correlations, we saw very similar pattern in the non-imputed and imputed data. We generally found non-significant correlations between the eye-tracking tasks, except for performance within the preferential looking task at 10 months and 18 months, see **Table S4**.

For the SE-CDI, the pattern is in general very similar between the imputed SE-CDI correlation matrix, and the non-imputed matrix. That is, strongly positive and significant associations in the non-imputed data are also strongly positive and significant in the imputed data. The exemption to this is the relation between SE-CDI at 11.5 months and SE-CDI word comprehension at 24 months. In the imputed data, we generally see moderate correlations between these measurements. For instance, we see that word comprehension at 10 months and word comprehension at 24 months correlate (rho = .47, *p* = .001), see Table 4 in the main text. However, in the non-imputed data, this correlation is at .14, and not significant. Thus, the relation is estimated to be much stronger in the imputed data. As we had a high attrition rate at 24 months, we note that the EM imputation should reasonably have the largest effect here. With a higher attrition rate, it is less clear how well the result represents our entire sample (compared to if we had a complete dataset). At the same time, this technique is superior to approaches such as deletion, mean-sub- station, and prior imputation approaches (e.g., Baraldi & Enders, 2010).

| **Table S3**  *Descriptive data showing median, min, max, skew and kurtosis for all primary outcome measures. Parentheses vales denote imputed values.* | | | | | |
| --- | --- | --- | --- | --- | --- |
| **Task** | **Median** | **Min** | **Max** | **Skew** | **Kurtosis** |
| **SE-SE-CDI**  ***Com. Vocabulary*** |  |  |  |  |  |
| 10 months | 22 (21.6) | 1 (-12) | 72 (72) | .68 (.55) | -.03 (.17) |
| 11.5 months | 38 (38) | 8 (-3) | 72 (92) | .11 (.29) | -1.21 (-.40) |
| 18 months | 70.5 (71.2) | 31 (23) | 86 (90) | -1.05 (-1.36) | 1.6 (2.90) |
| 24 months | 86 (86) | 58 (58) | 90 (98) | -2.24 (-1.66) | 7.18 (5.78) |
| ***Pro. Vocabulary*** |  |  |  |  |  |
| 10 months | 1 (1) | 0 (-5) | 16 (16) | 1.78 (1.66) | 2.96 (2.99) |
| 11.5 months | 3 (3) | 0 (-6) | 22 (28) | 1.6 (1.34) | 2.1 (2.02) |
| 18 months | 18 (20.5) | 1 (-42) | 86 (86) | 1.23 (.44) | .96 (1.38) |
| 24 months | 78 (75.5) | 1 (1) | 90 (96) | -1.54 (-1.36) | 1.88 (1.65) |
| **Preferential Looking Paradigm** |  |  |  |  |  |
| 10 months | .04 (.05) | -.17 (-.17) | .41 (.42) | .67 (.30) | .78 (.20) |
| 11.5 months | .02 (.02) | -.30 (-.30) | .31 (.31) | -.05 (-.07) | 1.14 (1.89) |
| 18 months | .13 (.11) | -.18 (-.26) | .45 (.45) | -.01 (-.16) | -.48 (-.07) |
| **Mismatch paradigm** |  |  |  |  |  |
| 10 months | .00 (-.02) | -.34 (-.34) | .30 (.35) | -.16 (.42) | .88 (1.65) |
| 11.5 months | -.03 (.01) | -.15 (-.19) | .31 (.34) | 1.22 (.85) | 1.51 (.35) |
| *Note.* SE-SE-CDI = A Swedish short form of the early vocabulary checklist; Com. vocabulary word comprehension sub-scale, Pro. vocabulary = word production sub-scale. | | | | | |

Finally, we looked at how the eye-tracking tasks relate to SE-SE-CDI. In general, we see the same pattern in the non-imputed data, such that when we see significant correlations in the imputed data, we also see moderate correlations in the non-imputed data (although not always significant). Again, we do see that the correlations to SE-CDI at 24 months are estimated to be stronger in the imputed data, compared to the non-imputed. For instance, in the imputed data, we see that performance within the mismatch task at 11.5 months have a strong correlation to word comprehension at 24 months (rho = -.38, *p*<.001). However, in the non-imputed data, this correlation is at -.27, and not significant.

As to further compare the imputed data with the non-imputed data, we also conducted a series of scatterplots on both the imputed and original dataset. These scatterplots are based on the significant correlations found between the eye-tracking tasks and SE-SE-CDI in our main analysis. Also see the scatterplots in **Figure S8- S21**. We note that the scatterplots show a similar pattern between our imputed and non-imputed dataset, indicating that the estimated correlations follow the same pattern as the original data. In turn, it lends credibility to our result.

| **Table S4.**  Correlations between the SE-CDI at 10, 11.5, 18 and 24 months and preferential looking task and mismatch task in the non-imputed data | | | | | | | | | | | | | |
| --- | --- | --- | --- | --- | --- | --- | --- | --- | --- | --- | --- | --- | --- |
| Variable | Com 10 | Pro 10 | Com 11.5 | Pro 11.5 | Com 18 | Pro 18 | Com 24 | Pro 24 | PLP 10 | PLP 11.5 | PLP 18 | MM10 | MM 11.5 |
| Com 10 |  | .59 *** | .81 *** | .43 *** | .56 *** | .39 ** | .30 * | .02 | -.22 | .11 | -.28 ¤ | .02 | -.20 |
| Pro 10 |  |  | .54 *** | .73 *** | .45 *** | .45 *** | .18 | .10 | -.20 | .01 | -.15 | -.10 | -.14 |
| Com 11.5 |  |  |  | .50 *** | .59 *** | .35 * | .14 | .06 | -.15 | .08 | -.11 | -.04 | -.32 |
| Pro 11.5 |  |  |  |  | .29 * | .22 | .13 | .01 | -.25 | -.01 | -.29 ¤ | -.07 | -.17 |
| Com 18 |  |  |  |  |  | .71 *** | .73 *** | .40 ** | .05 | .22 | .17 | -.17 | -.18 |
| Pro 18 |  |  |  |  |  |  | .70 *** | .57 *** | .05 | .16 | .17 | -.08 | -.24 |
| Com 24 |  |  |  |  |  |  |  | .58 *** | -.01 | .06 | .06 | -.26 | -.27 |
| Pro 24 |  |  |  |  |  |  |  |  | -.03 | -.03 | .13 | -.06 | -.36 |
| PLP 10 |  |  |  |  |  |  |  |  |  | -.17 | .30 | -.01 | .05 |
| PLP 11.5 |  |  |  |  |  |  |  |  |  |  | .02 | -.06 | -.02 |
| PLP 18 |  |  |  |  |  |  |  |  |  |  |  | -.12 | -.16 |
| MM 10 |  |  |  |  |  |  |  |  |  |  |  |  | -.14 |
| MM 11.5 |  |  |  |  |  |  |  |  |  |  |  |  |  |

*Note.* Com = SE-SE-CDI word comprehension. Pro = SE-SE-CDI word production. PLP = preferential looking paradigm. MM = mismatch paradigm. *, *p* < .05, **, *p* < .01, ***, *p* <.001, ¤ borderline significant (*p* = .051-.056).


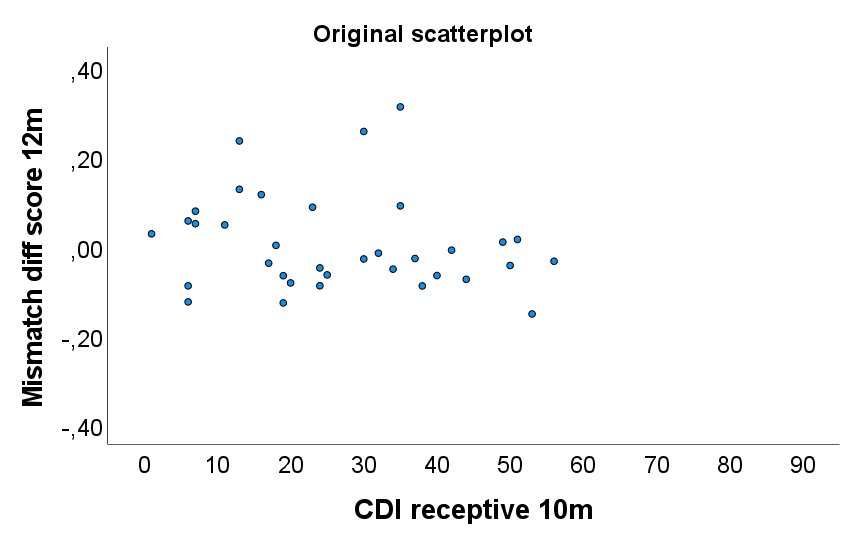

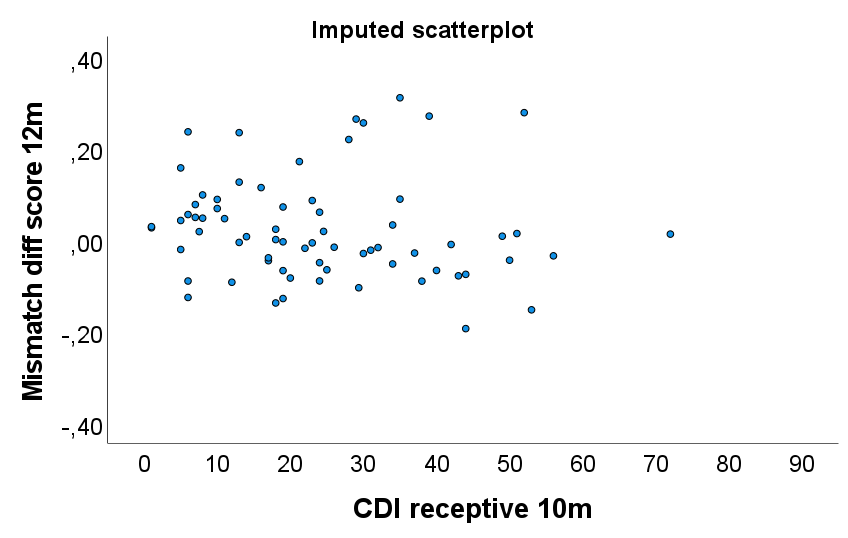
**Figure S8.**

Scatterplots of the Mismatch difference score at 11.5 months and SE-CDI word comprehension subscale at 10 months, for the original (left) and imputed (right) dataset, respectively.


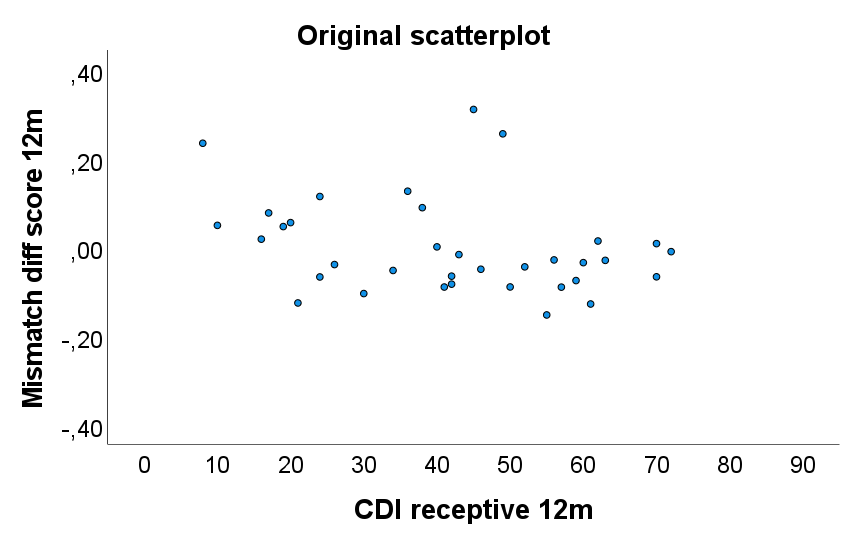

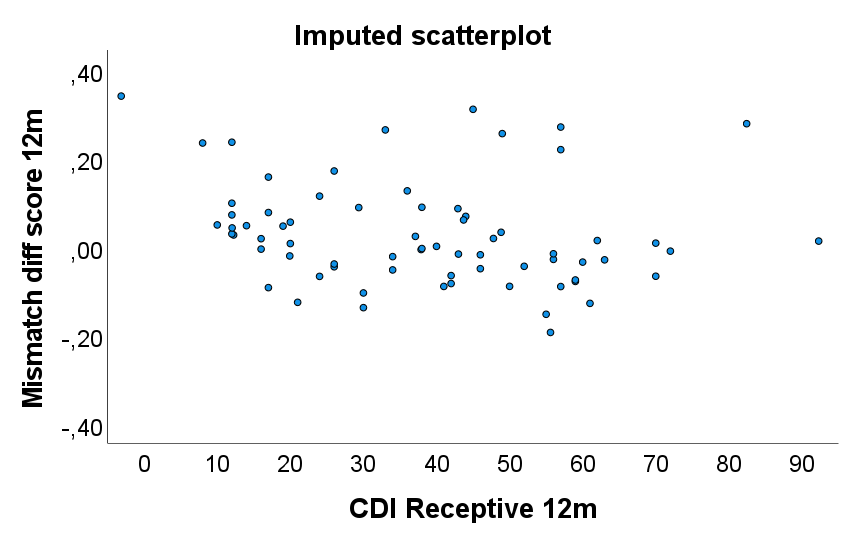


**Figure S9.**

Scatterplots of the Mismatch difference score at 11.5 months and SE-CDI word comprehension subscale at 11.5 months, for the original (left) and imputed (right) dataset, respectively.


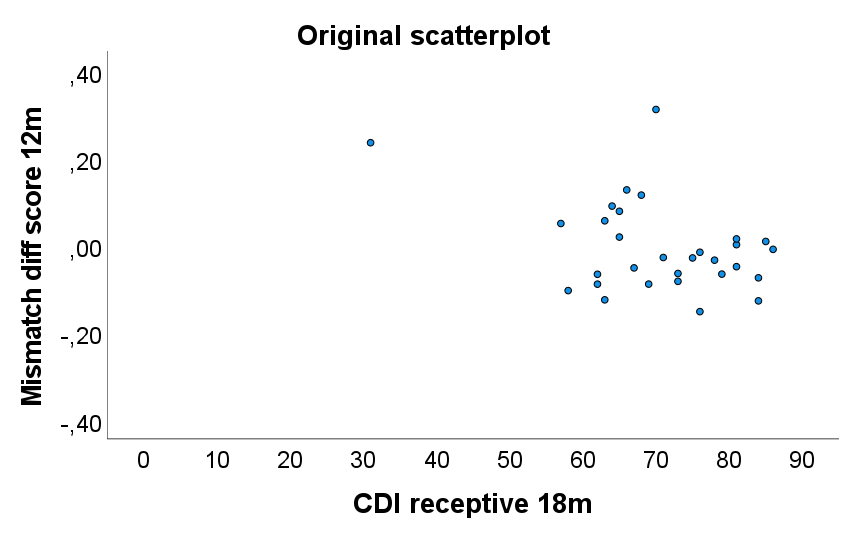

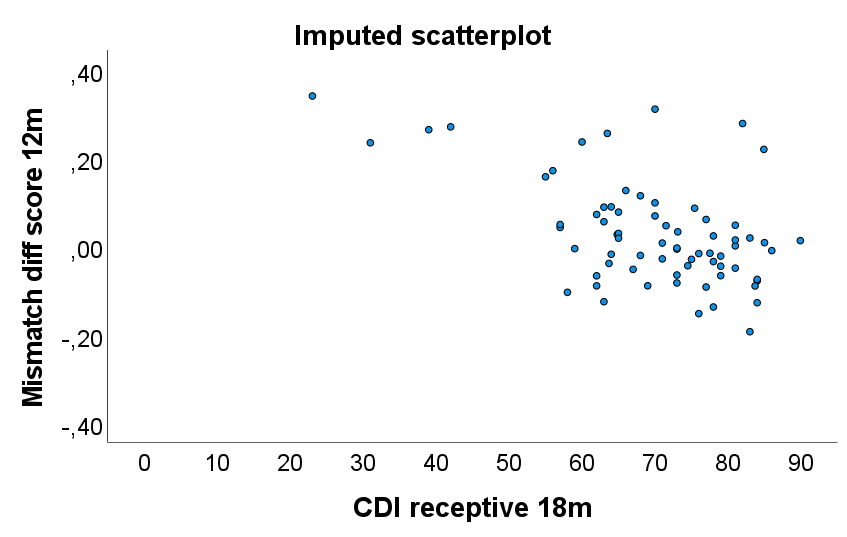


**Figure S10.**

Scatterplots of the Mismatch difference score at 11.5 months and SE-CDI word comprehension subscale at 18 months, for the original (left) and imputed (right) dataset, respectively.


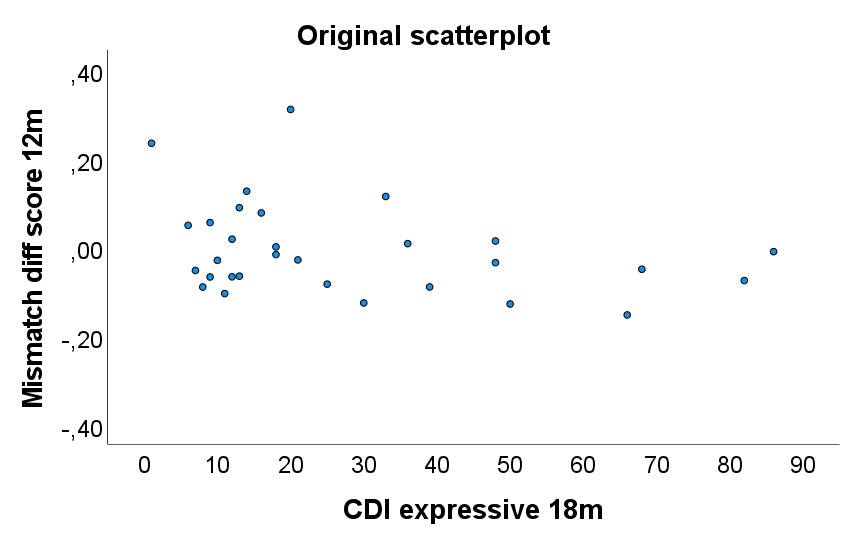

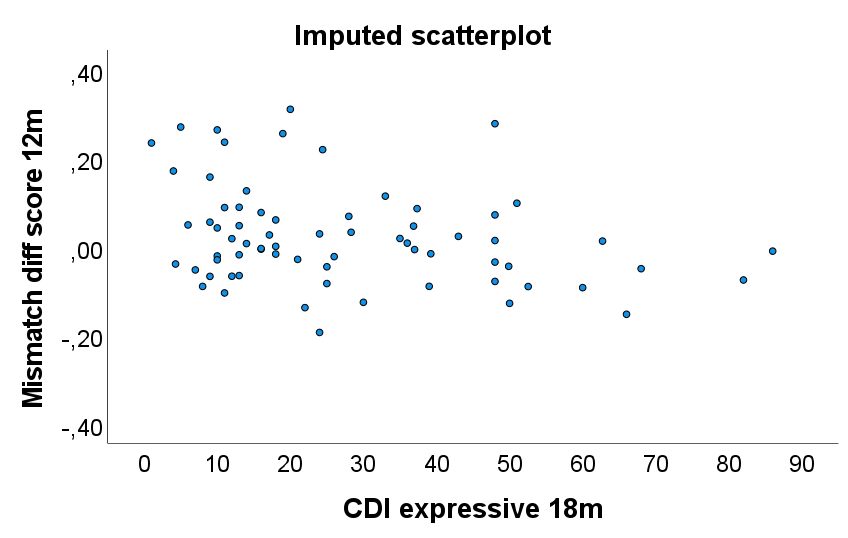


**Figure S11.**

Scatterplots of the Mismatch difference score at 11.5 months and SE-CDI word production subscale at 18 months, for the original (left) and imputed (right) dataset, respectively.


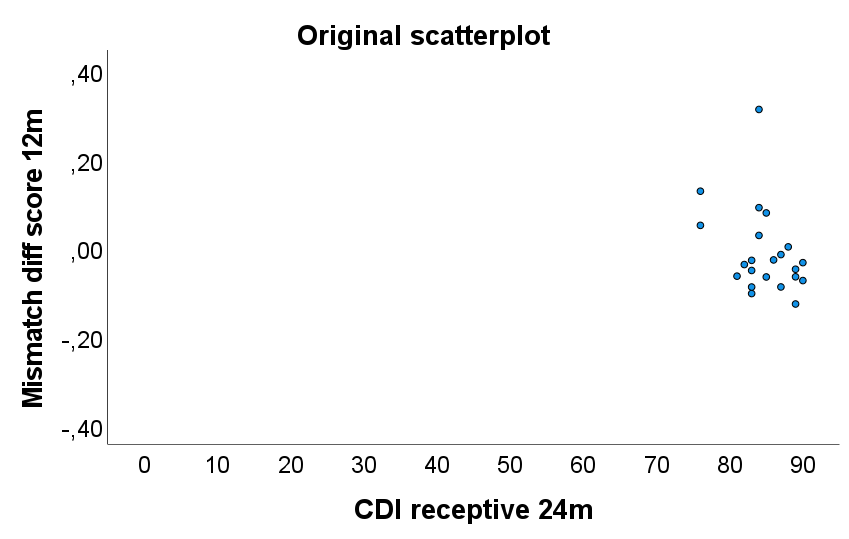

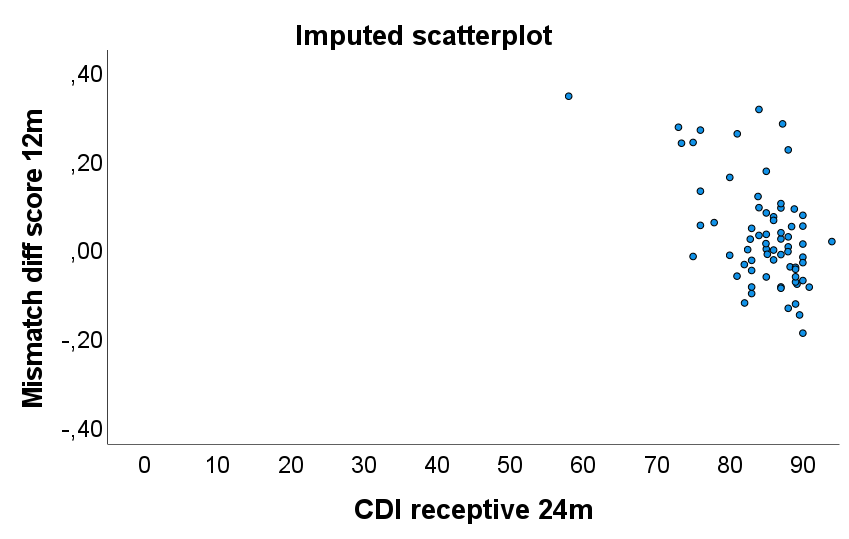


***Figure S12****.*

Scatterplots of the Mismatch difference score at 11.5 months and SE-CDI word comprehension subscale at 24 months, for the original (left) and imputed (right) dataset, respectively.


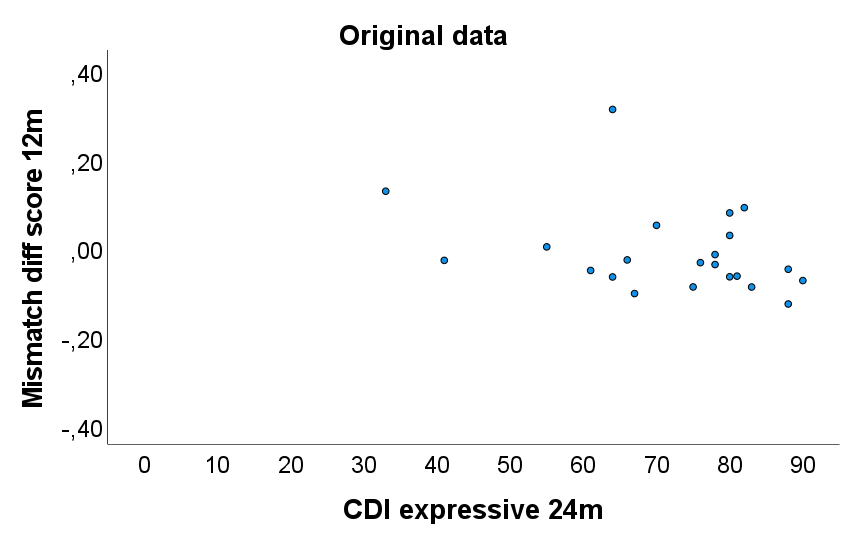

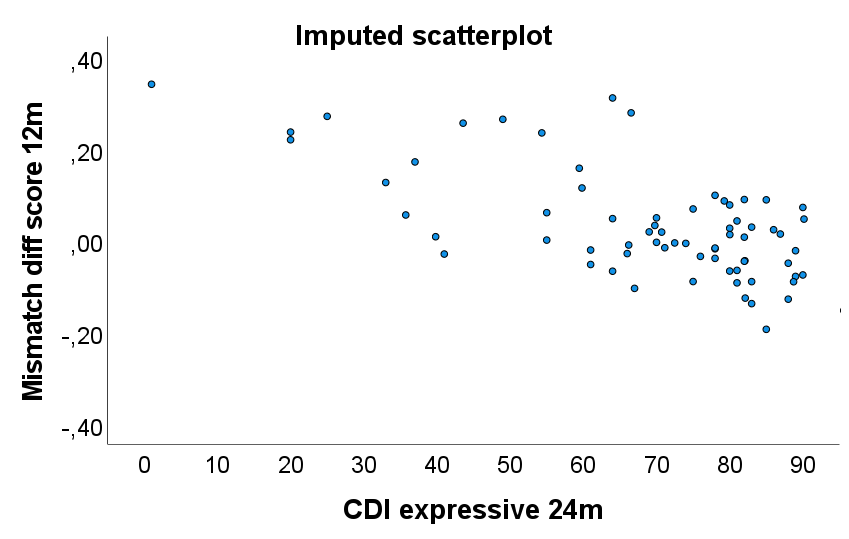


**Figure S13.**

Scatterplots of the Mismatch difference score at 11.5 months and SE-CDI word production subscale at 24 months, for the original (left) and imputed (right) dataset, respectively.


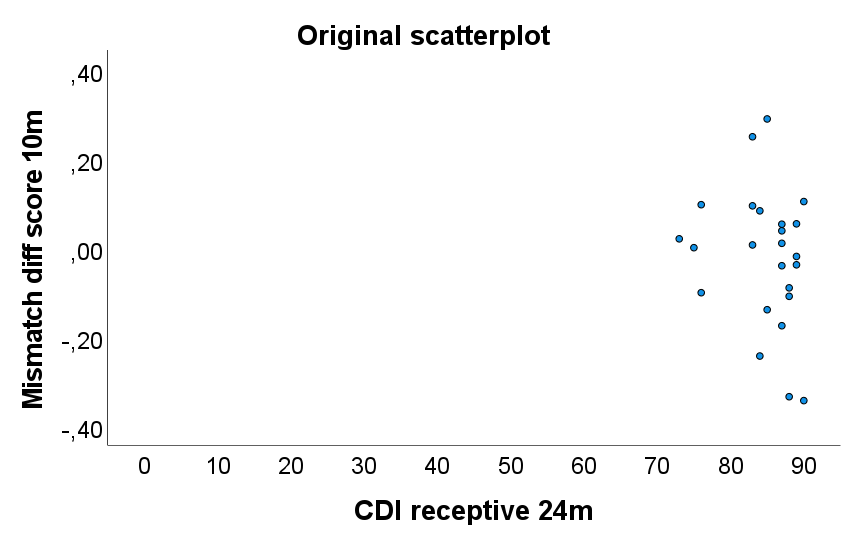

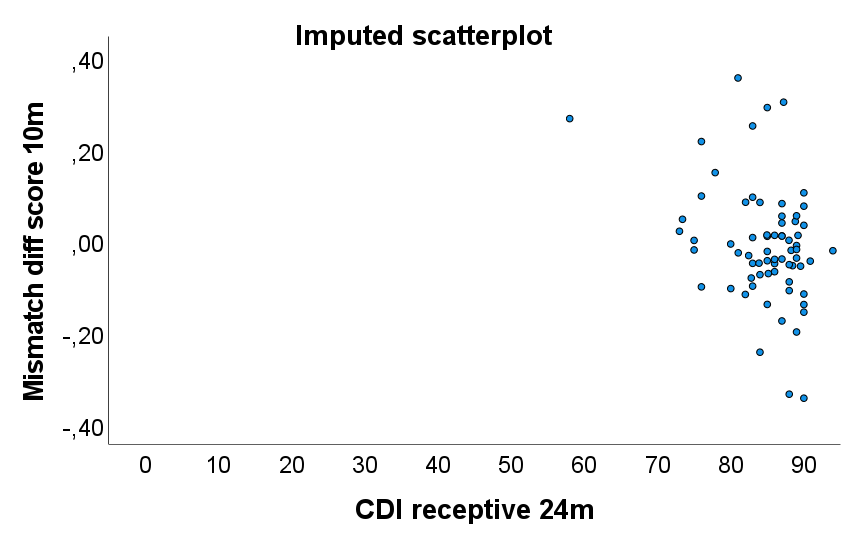


**Figure S14.**

Scatterplots of the Mismatch difference score at 10 months and SE-CDI word comprehension subscale at 24 months, for the original (left) and imputed (right) dataset, respectively.


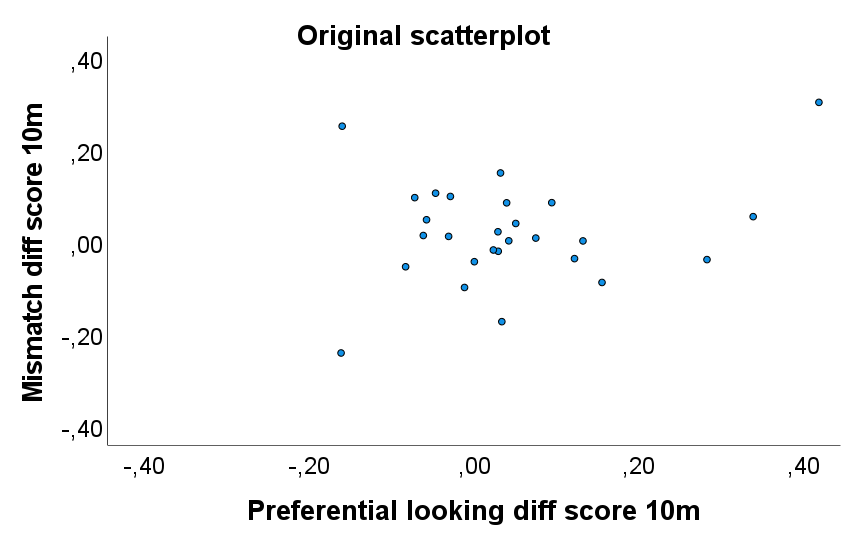

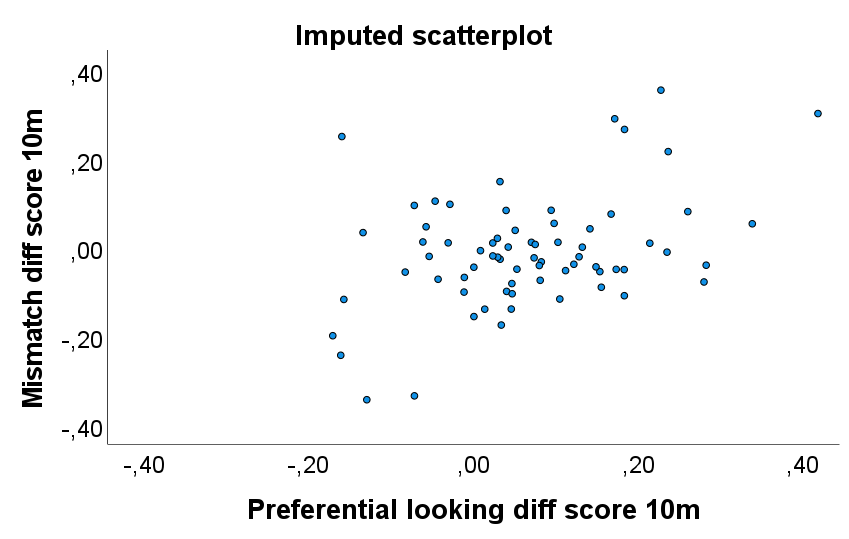


**Figure S15.**

Scatterplots of the Mismatch difference score at 10 months and preferential looking difference score at 10 months, for the original (left) and imputed (right) dataset, respectively.


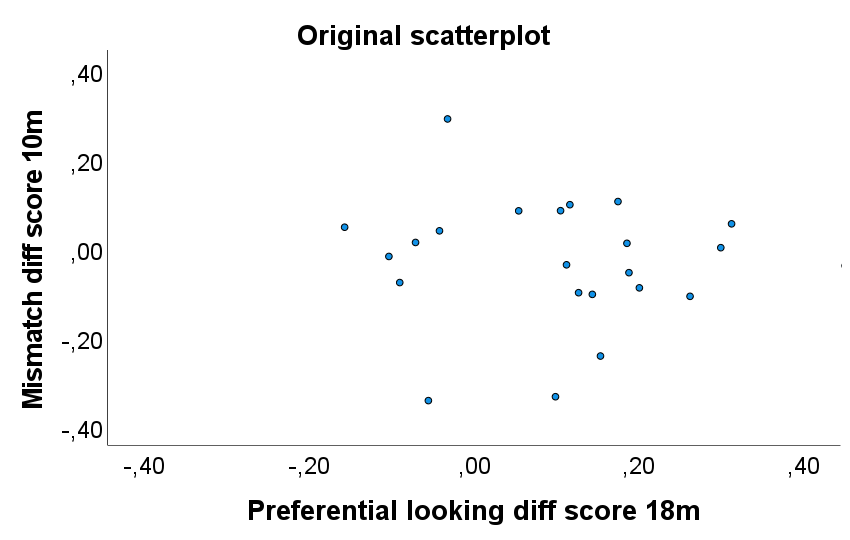

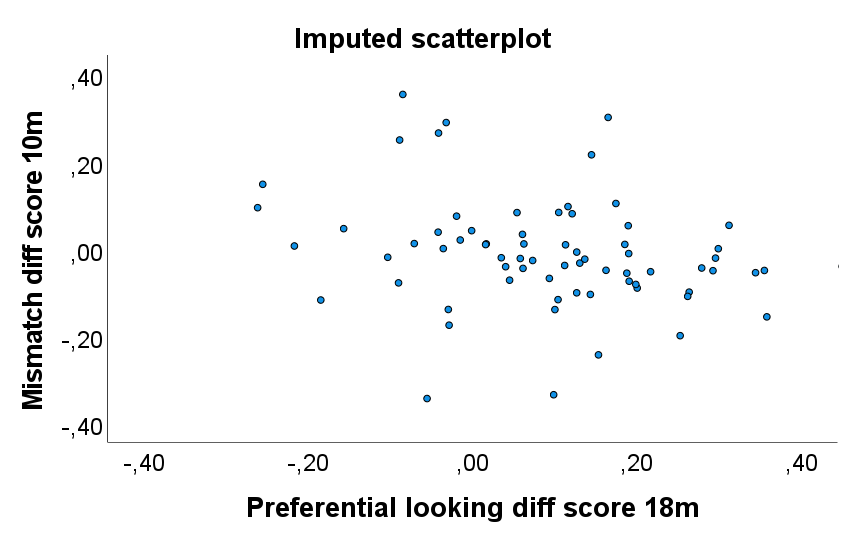


**Figure S16.**

Scatterplots of the Mismatch difference score at 10 months and preferential looking difference score at 18 months, for the original (left) and imputed (right) dataset, respectively.


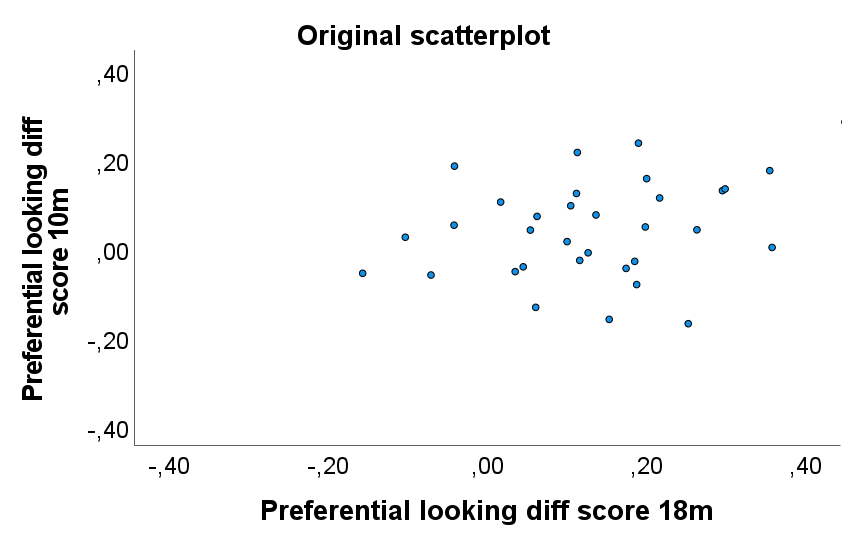

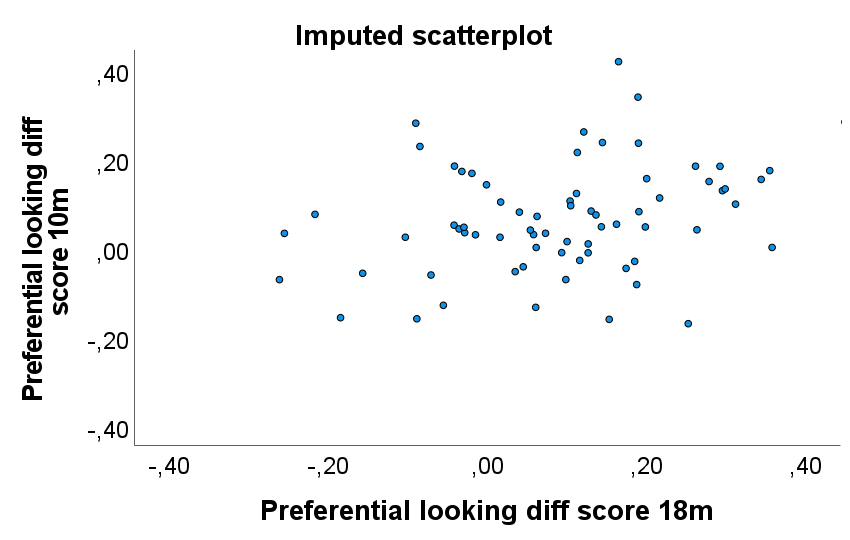


**Figure S17.**

Scatterplots of the preferential looking difference score at 10 months and the preferential looking difference score at 18 months, for the original (left) and imputed (right) dataset, respectively


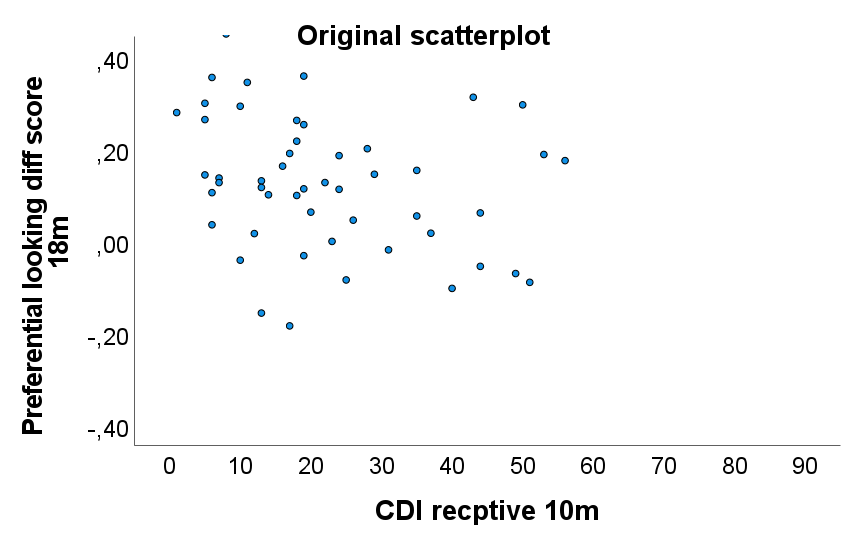

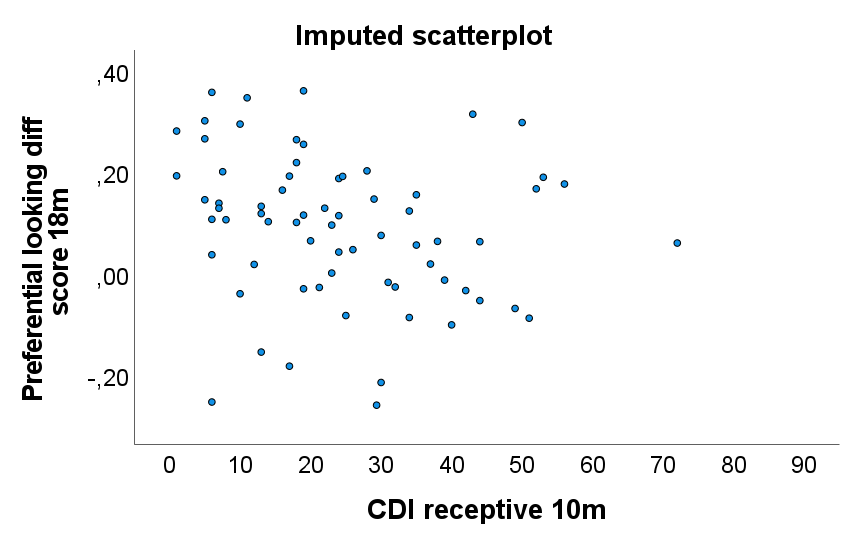


**Figure S18.**

Scatterplots of the preferential looking difference score at 18 months and SE-CDI word comprehension subscale at 10 months, for the original (left) and imputed (right) dataset, respectively.


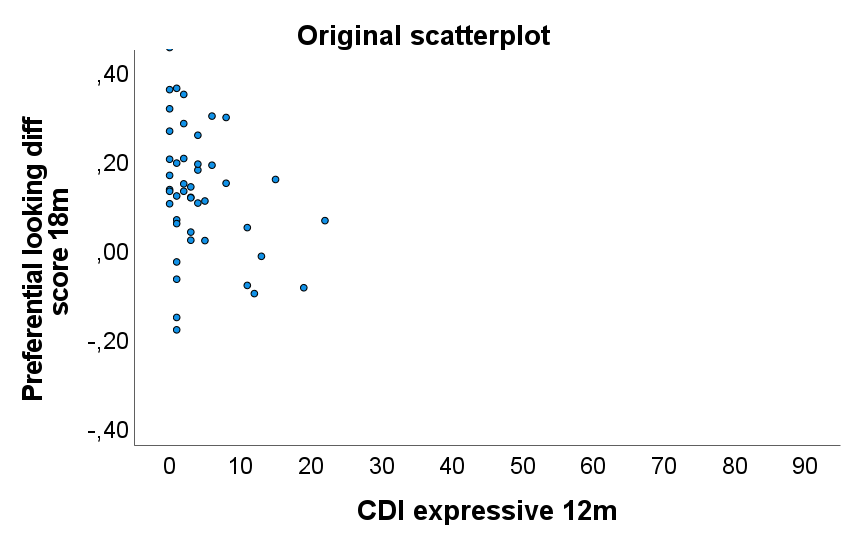

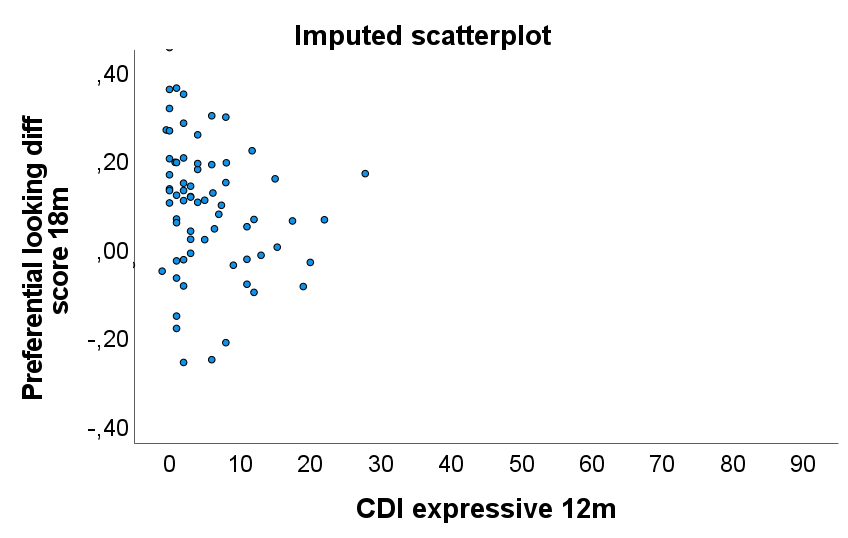


**Figure S19**.

Scatterplots of the preferential looking difference score at 18 months and SE-CDI word production subscale at 11.5 months, for the original (left) and imputed (right) dataset, respectively.


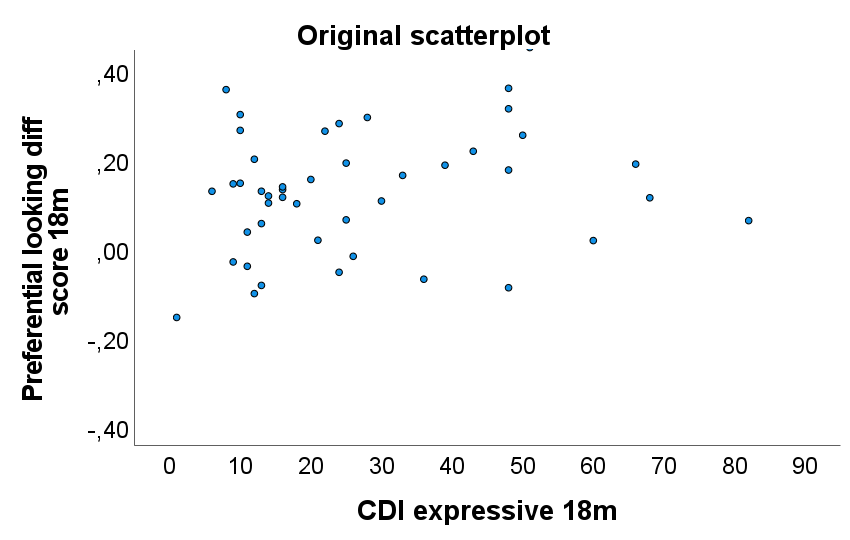

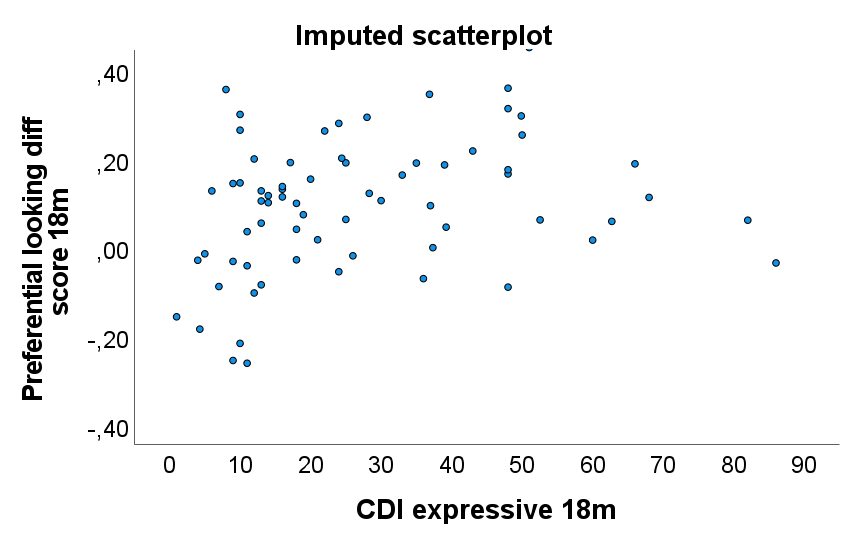


**Figure S20**.

Scatterplots of the preferential looking difference score at 18 months and SE-CDI word production subscale at 18 months, for the original (left) and imputed (right) dataset, respectively.


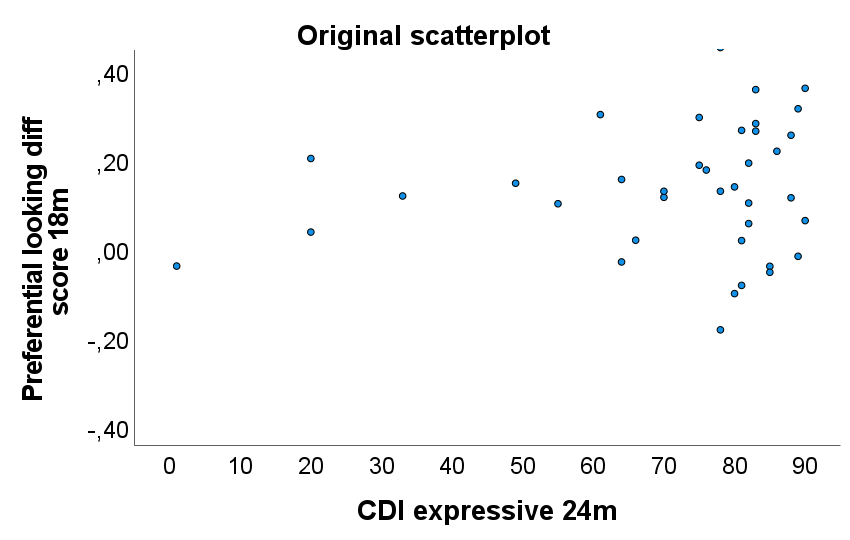

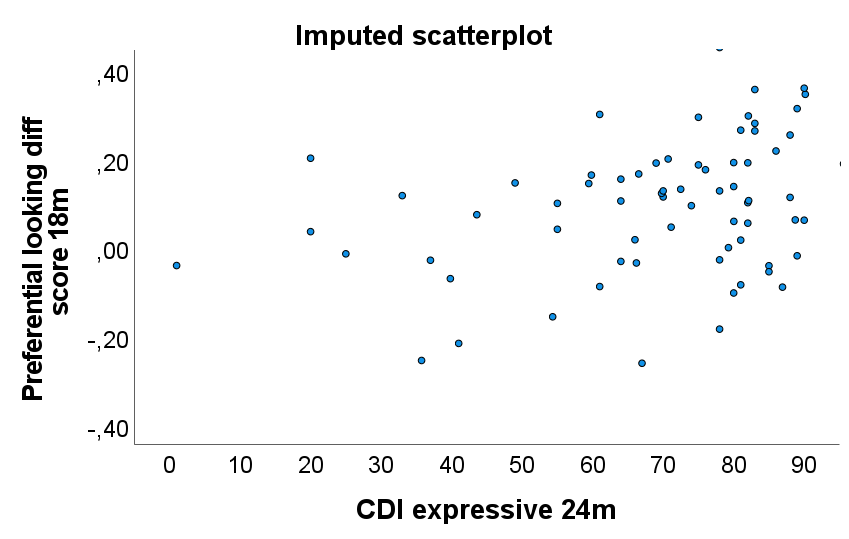


**Figure S21.**

Scatterplots of the preferential looking difference score at 18 months and SE-CDI word production subscale at 24 months, for the original (left) and imputed (right) dataset, respectively.

**Controlling for Socio-Demographic Variables within the SE-SE-CDI**

To explore whether socio-demographic factors influenced SE-SE-CDI correlations, Table S5 presents correlation matrices. While some correlation strengths varied slightly, the direction and significance levels remained unchanged, indicating that parental vocabulary estimates were not substantially affected by environmental factors.

| **Table S5.**  *Spearman Correlations between the SE-SE-CDI (at all ages) and Preferential Looking and Mismatch Paradigms when Controlling for Socio-Demographic Variables.* | | | | | |
| --- | --- | --- | --- | --- | --- |
| **Variable** | **PLP**  **10 months** | **PLP**  **11.5 months** | **PLP**  **18 months** | **MM**  **10 months** | **MM**  **11.5 months** |
| ***SE-SE-CDI*** |  |  |  |  |  |
| Comprehension 10 | -.12 | .16 | -.26 | .23 | -.25 |
| Production 10 | -.03 | .08 | -.07 | -.02 | -.11 |
| Comprehension 11.5 | -.07 | .16 | -.13 | .14 | -.34 * |
| Production 11.5 | .11 | .06 | -.30 * | .24 | .03 |
| Comprehension 18 | .05 | .25 | .01 | -.06 | -.44 *** |
| Production 18 | .06 | .14 | .30* | -.02 | -.44 *** |
| Comprehension 24 | .11 | .17 | .16 | -.18 | -.39 ** |
| Production 24 | .15 | .03 | .39 ** | -.15 | -.51 *** |
| ***Word Recognition*** |  |  |  |  |  |
| PLP 10 |  | -.19 | .28 * | .25 | .13 |
| PLP 11.5 |  |  | .05 | -.10 | -.03 |
| PLP 18 |  |  |  | -.31 * | -.01 |
| MM 10 |  |  |  |  | -.10 |
| MM 11.5 |  |  |  |  |  |
| Note. Comprehension = SE-SE-CDI word comprehension. Production = SE-SE-CDI word production. PLP = preferential looking paradigm. MM = Mismatch paradigm. *, *p* < .05, **, *p* < .01, ***, *p* <.001. | | | | | |

**References**

Baraldi, A. N., & Enders, C. K. (2010). An introduction to modern missing data analyses. *Journal of school psychology*, *48*(1), 5-37.

Bergelson, E., & Swingley, D. (2012). At 6–9 months, human infants know the meanings of many common nouns. *Proceedings of the National Academy of Sciences*, *109*(9), 3253-3258.

Bergelson, E., & Swingley, D. (2015). Early word comprehension in infants: Replication and extension. *Language Learning and Development*, *11*(4), 369-380.

Eriksson, M., Westerlund, M., & Berglund, E. (2002). A screening version of the Swedish communicative development inventories designed for use with 18-month-old children.

Fenson, L., Dale, P. S., Reznick, J. S., Bates, E., Thal, D. J., Pethick, S. J., ... & Stiles, J. (1994). Variability in early communicative development. *Monographs of the society for research in child development*, i-185.

Fenson, L., Pethick, S., Renda, C., Cox, J. L., Dale, P. S., & Reznick, J. S. (2000). Short-form versions of the MacArthur communicative development inventories. *Applied psycholinguistics*, *21*(1), 95-116.

Nyström, P., Falck-Ytter, T., & Gredebäck, G. (2016). The TimeStudio Project: An open source scientific workflow system for the behavioral and brain sciences. *Behavior research methods*, *48*(2), 542-552.

Parise, E., & Csibra, G. (2012). Electrophysiological evidence for the understanding of maternal speech by 9-month-old infants. *Psychological science*, *23*(7), 728-733.
